# High‐density genetic map using whole‐genome resequencing for fine mapping and candidate gene discovery for disease resistance in peanut

**DOI:** 10.1111/pbi.12930

**Published:** 2018-05-15

**Authors:** Gaurav Agarwal, Josh Clevenger, Manish K. Pandey, Hui Wang, Yaduru Shasidhar, Ye Chu, Jake C. Fountain, Divya Choudhary, Albert K. Culbreath, Xin Liu, Guodong Huang, Xingjun Wang, Rupesh Deshmukh, C. Corley Holbrook, David J. Bertioli, Peggy Ozias‐Akins, Scott A. Jackson, Rajeev K. Varshney, Baozhu Guo

**Affiliations:** ^1^ Crop Protection and Management Research Unit USDA‐ARS Tifton GA USA; ^2^ Department of Plant Pathology University of Georgia Tifton GA USA; ^3^ Center of Excellence in Genomics & Systems Biology International Crops Research Institute for the Semi‐Arid Tropics (ICRISAT) Hyderabad India; ^4^ Center for Applied Genetic Technologies Mars Wrigley Confectionery Athens GA USA; ^5^ Center for Applied Genetic Technologies University of Georgia Athens GA USA; ^6^ Department of Horticulture and Institute of Plant Breeding & Genomics University of Georgia Tifton GA USA; ^7^ BGI‐Shenzhen Shenzhen China; ^8^ Shandong Academy of Agricultural Sciences Biotechnology Research Center Jinan China; ^9^ University Laval Quebec Canada; ^10^ Crop Genetics and Breeding Research Unit USDA‐ARS Tifton GA USA

**Keywords:** whole‐genome resequencing, high‐density genetic map, quantitative trait loci, early leaf spot, late leaf spot, Tomato spotted wilt virus

## Abstract

Whole‐genome resequencing (WGRS) of mapping populations has facilitated development of high‐density genetic maps essential for fine mapping and candidate gene discovery for traits of interest in crop species. Leaf spots, including early leaf spot (ELS) and late leaf spot (LLS), and Tomato spotted wilt virus (TSWV) are devastating diseases in peanut causing significant yield loss. We generated WGRS data on a recombinant inbred line population, developed a SNP‐based high‐density genetic map, and conducted fine mapping, candidate gene discovery and marker validation for ELS, LLS and TSWV. The first sequence‐based high‐density map was constructed with 8869 SNPs assigned to 20 linkage groups, representing 20 chromosomes, for the ‘T’ population (Tifrunner × GT‐C20) with a map length of 3120 cM and an average distance of 1.45 cM. The quantitative trait locus (QTL) analysis using high‐density genetic map and multiple season phenotyping data identified 35 main‐effect QTLs with phenotypic variation explained (PVE) from 6.32% to 47.63%. Among major‐effect QTLs mapped, there were two QTLs for ELS on B05 with 47.42% PVE and B03 with 47.38% PVE, two QTLs for LLS on A05 with 47.63% and B03 with 34.03% PVE and one QTL for TSWV on B09 with 40.71% PVE. The epistasis and environment interaction analyses identified significant environmental effects on these traits. The identified QTL regions had disease resistance genes including R‐genes and transcription factors. KASP markers were developed for major QTLs and validated in the population and are ready for further deployment in genomics‐assisted breeding in peanut.

## Introduction

Peanut, *Arachis hypogaea* (2*n =* 4*x =* 40), is an allotetraploid with an AABB genomic constitution which originated from a single recent hybridization and spontaneous tetraploidization of two ancestral diploid species, *A. duranensis* (A‐genome) and *A. ipaensis* (B‐genome) approximately 4000–6000 years ago (Bertioli *et al*., 2016; Halward *et al*., 1992; Moretzsohn *et al*., 2013). The relatively short evolutionary history of domesticated peanut and the presence of hybridization barriers between diploid and tetraploid species has been one of the major reasons for the narrow genetic base in cultivated peanuts. This low level of diversity has been a major hurdle for mining large‐scale polymorphic markers, such as simple‐sequence repeats (SSRs) for construction of high‐density genetic maps and QTL studies (Pandey *et al*., 2016; Qin *et al*., 2012; Varshney *et al*., 2013). The genetic yield potential of peanut cultivars has been continuously challenged by several diseases including early leaf spot (ELS) caused by *Cercospora arachidicola*, late leaf spot (LLS) caused by *Cercosporidium personatum* and *Tomato spotted wilt virus* (TSWV). These foliar diseases cause yield losses of up to 70%, resulting in approximately $600 million in losses (Food and Agriculture Organization (FAO), 2004; Ogwulumba *et al*., 2008). While insecticides and fungicides have been used as part of an integrated pest management approach, breeding disease‐resistant cultivars with high yield and good agronomic performance is the most economical and sustainable solution (Guo *et al*., 2013; Pandey *et al*., 2012; Varshney *et al*., 2013).

Genomics‐assisted breeding (GAB) has demonstrated great potential for accelerated development of improved varieties (Varshney *et al*., 2009) including peanut. However, it is worth mentioning that success of the diagnostic markers in breeding depends on the precise phenotyping and high‐density genotyping. The earlier efforts towards identifying linked markers for leaf spot and TSWV resistance were based on SSR‐based genetic maps using T‐population (Tifrunner × GT‐C20) (Pandey *et al*., 2014, 2017a; Qin *et al*., 2012; Wang *et al*., 2012). After making use of available SSR resources in the public domain, a total of 418 SSR loci could be mapped leading to identification of several QTLs with <20% phenotypic variation explained (PVE) for these diseases (Pandey *et al*., 2017a). The identified genomic regions were also too large to be exploited in diagnostic marker development.

The last decade has witnessed rapid progress in genome sequencing that greatly helped in high‐resolution trait mapping, candidate gene discovery and breeding applications in many legumes (Pandey *et al*., 2016). Single nucleotide polymorphisms (SNPs) have high frequency of occurrence throughout the genome and are genetic markers of choice for several genetic and breeding applications. Recent availability of reference genomes for both the progenitor species, namely, *A. duranensis* (Bertioli *et al*., 2016; Chen *et al*., 2016) and *A. ipaensis* (Bertioli *et al*., 2016), has made the application of next‐generation sequencing (NGS) approach a possibility in peanut. Bertioli *et al*. (2016) not only provided insights on genome architecture and evolution but also opened opportunities for developing other required genomic tools and technologies for deployment in different genetic and breeding applications. For example, these reference genomes allowed for the construction of a high‐density genotyping array with >58K highly informative SNPs (Pandey *et al*., 2017b) and SNP identification using RNA‐seq data (Nayak *et al*., 2017), which further opens an array of options for deeper exploration of genome and gene discovery. Further, the accelerated pace of developments in sequencing technologies has made sequencing more affordable due to increased throughput data generation and competition in the market.

We resequenced the parental genotypes and the RIL population for conducting high‐resolution genetic mapping and development of diagnostic markers for resistance to leaf spots and TSWV for peanut breeding application. The detailed sequence analysis of a RIL population has facilitated QTL discovery for both the diseases followed by candidate gene discovery and marker identification. This study also developed and validated PCR‐based KASP (kompetitive allele‐specific polymerase chain reaction) markers which can now be deployed in marker‐assisted selection. This study also provided the first SNP‐based high‐density genetic map currently available for cultivated peanut which can be used for a variety of applications including the improvement of tetraploid peanut reference genome assemblies.

## Results

### Variation in disease severity in parents and RIL population

The evaluation of disease resistance was all conducted in the field based on natural infection. We did not experience any interactions among these three diseases. As the causal organism is different for each disease, there were no any studies to investigate the possible interaction among these diseases. The distributions of the ratings of disease severity for ELS, LLS and TSWV were relatively normal except a few instances, where some individuals of the population showed extreme phenotypes and were out of the normal curve (Figure S1). Overall, the quantitative nature of the investigated traits was observed. The phenotypes for disease severity ratings of two parental genotypes and RILs showed significant differences among environments across the environments (associated with major QTLs) (Table S1). Disease severity of ELS for the parental lines ranged from 2.3 to 3.3 for ‘Tifrunner’ and from 3.8 to 7.0 for GT‐C20 during 2009–2013. ELS severity in RILs ranged from 1.0 to 8.0 over the same period. In case of LLS, disease ratings for Tifrunner ranged from 5.0 to 6.5, 7.2 to 10.0 for GT‐C20 and 4.7 to 10.0 among RILs over the same period. TSWV severity in the parents ranged from 1.7 to 2.3 for Tifrunner and 4.7 to 5.7 for GT‐C20, while the disease severity among RILs ranged from 1.0 to 6.7 (Figure S1).

### Sequencing of the RIL population and SNP discovery

Over one Tb of filtered data (~23 billion reads) was generated for the parental lines and 91 selected RILs. The first parent, Tifrunner, was sequenced at 100X and the second parent, GT‐C20, at 10X coverage, while the RIL population individuals were each sequenced at 2–5X coverage (Table S2). After quality filtering, an average read length of 93, 96 and 97 bp was obtained for Tifrunner, GT‐C20 and individual RILs, respectively. In total, ~40% of sequence data were mapped on A‐subgenome and ~60% on B‐subgenome except for line T92 where more than 60% of reads were mapped on A‐subgenome. Although the amount of data produced from each plant sample was different, the proportion of mapped reads on the respective genomes was similar for each of the individual plants with the exception of T92 (Figure S2). It could be due to the exchanges between A‐ and B‐subgenomes, as reported by Leal‐Bertioli *et al*. (2015) that autotetraploid‐like tetrasomic recombination is quite frequent in RIL population derived from a cultivated peanut. As Tifrunner has considerable tetrasomic regions and the RIL population with Tifrunner as a parental line could observe these changes in the progeny. All the reads mapped to the reference genome were used for haplotype‐based SNP calling. A total of 97 571 SNPs were detected between the two parental lines, and 18 252 of those remained after removing the low‐quality SNPs (Table S3). The SNPs were distributed throughout the 20 linkage groups (LGs) with the highest number of SNPs occurring on chromosome A06 (2771) and the fewest on chromosome B07 (261). Noneven distribution of SNPs on the genomes could also be attributed to the presence of autotetraploid‐like tetrasomic regions (either AAAA or BBBB) in the genomes, where the chances of finding the SNPs are almost negligible (Clevenger *et al*., 2017; Leal‐Bertioli *et al*., 2015). RILs thus obtained from Tifrunner as one of the parent are expected to have lesser or no markers identified in the regions with tetrasomy. Of the 18 252 high‐quality SNPs, 16 674 SNPs could genotype the population and were polymorphic. The other 1578 SNPs could not be genotyped in the population, which could be due to the errors in sequencing of those parental genotypes or difference in sequence depth of the parents and the RILs. One of the parents, Tifrunner, was sequenced at 100× depth, and GT‐C20 was at about 10X. However, the RILs were sequenced at much lower depth (2X to 5X). Of these, 10 274 SNPs showed less than 20% missing data and no segregation distortion, and were used for genetic mapping (Figure S3).

### Most dense genetic map with homeologous and translocated markers

Of the 10 274 SNP markers obtained using haplotype SNP mining, 8869 SNPs were mapped onto 20 LGs spanning a genetic map length of 3120.71 cM with map density of 1.45 cM/locus. Many SNPs occupied the same genetic loci on the LGs; therefore, a total of 2156 marker loci covering 8869 SNPs were mapped on the 20 LGs (Figure S4). The mapped marker loci per LG ranged from 38 (B07) to 179 (A03) with an average of 107.8 loci/LG. A‐ and B‐genome LGs were identified with 1219 marker loci covering 1637.8 cM (1.34 avg. marker distance) and 937 marker loci with 1484.91 cM genetic map distance (1.58 avg. marker distance), respectively (Table 1). Overall, the SNP markers were usually densely and homogeneously distributed along the 20 LGs except gaps of more than 10 cM each on LGs A01, A07, A08, B01 and B08, and five such gaps on B07 between two contiguous markers.

**Table 1:** Summary of total number of SNP markers across 20 linkage groups

Linkage groups	Total markers on map	A‐markers	B‐markers	Translocated markers	Av. Interval (cM)	Length (cM)	Loci
		/Homeologous	/Homeologous				
A01	278	260	13	5	1.48	176.02	119
A02	346	296	27	23	1.3	185.7	143
A03	1222	1047	76	99	1.08	192.46	179
A04	676	623	33	20	1.43	153.52	107
A05	701	647	36	18	1.22	190.24	156
A06	1644	1529	46	69	1.24	157.13	127
A07	406	368	15	23	1.76	89.59	51
A08	244	210	13	21	1.54	181.7	118
A09	475	426	33	16	1.47	154.56	105
A10	307	267	25	15	1.38	156.88	114
B01	304	29	272	3	1.78	153.18	86
B02	311	37	260	14	1.37	143.5	105
B03	373	153	215	5	1.29	175.91	136
B04	164	26	134	4	1.5	136.25	91
B05	334	40	282	12	1.36	142.86	105
B06	283	41	237	5	1.58	163.98	104
B07	50	6	35	9	4.28	162.58	38
B08	159	8	135	16	1.5	85.54	57
B09	252	28	204	20	1.51	173.3	115
B10	340	54	270	16	1.46	145.81	100
Total/average	8869			413	1.45	3120.71	2156

Highlighted in grey are the homeologous markers on the LGs of A‐ and B‐subgenomes.

**Figure 1:**
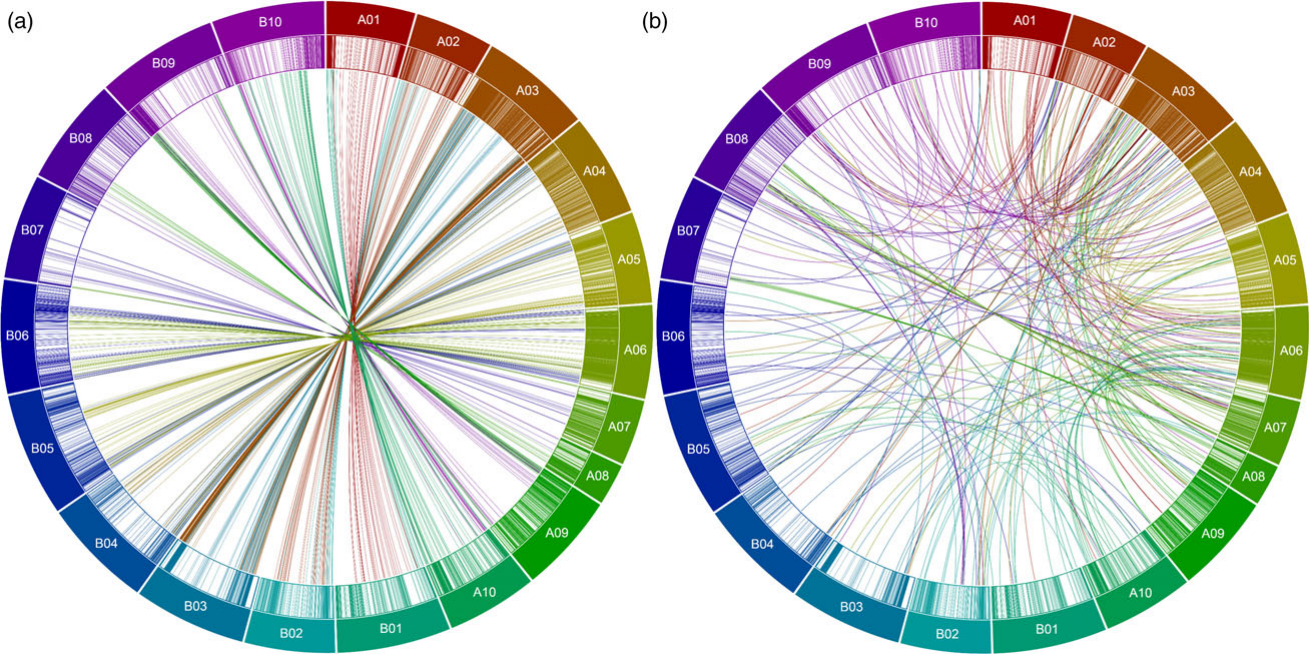
Circos plots showing the distribution of SNPs. (a) Homeologous SNPs: A01‐A10 represent A‐subgenome chromosomes, and corresponding originating threads represent SNPs identified on A‐chromosome but mapped on corresponding B‐LGs and vice versa for B01‐B10. (b) Translocated SNPs: SNPs identified on A‐ or B‐chromosomes but mapped on other chromosomes of either subgenome except for their homeologous (corresponding) chromosomes. Different coloured blocks represent the different LGs. Connecting coloured lines represent the homeologous and translocated SNP movement across various LGs based on the obtained LGs.

Homeologous and translocated SNP markers were also identified in the current genetic map. A total of 422 homeologous markers identified from A‐subgenome were mapped on B‐subgenome. Similarly, 317 homeologous SNPs from B‐subgenome were mapped on the A‐subgenome (Table 1, Figure 1a, b). A total of 309 and 104 translocated markers were identified on LGs A01‐A10 and B01‐B10, respectively. For example, LGs A03 and A06 have 99 (68 from A‐chromosomes; 31 from B‐chromosomes) and 69 (45 from A‐chromosomes; 25 from B‐chromosomes) translocated markers, respectively (Tables 1, S4). Largely, the markers were seen to have been moved from the end of each chromosome to corresponding and other LGs (Figure 1a, b).

We also considered the major QTL (PVE ≥ 10%) defining SSR markers reported in our earlier study (Pandey *et al*., 2017a) in the current map. Of the 19 major QTL‐linked markers reported earlier, 15 were mapped to the current LGs. However, integration of these markers increased the genetic distance of the respective LGs (Table S5). Thirteen of these 15 markers were mapped to the same LGs as previously reported, but remaining two markers were mapped to LG B03 instead of A03 as reported earlier. The two SSR loci (TC38F01, GM1986‐2) on A07 were the first and the last on that linkage group, and all the SNPs identified in the current study were harboured between these two SSR loci.

### QTLs associated with disease resistance

**Figure 2:**
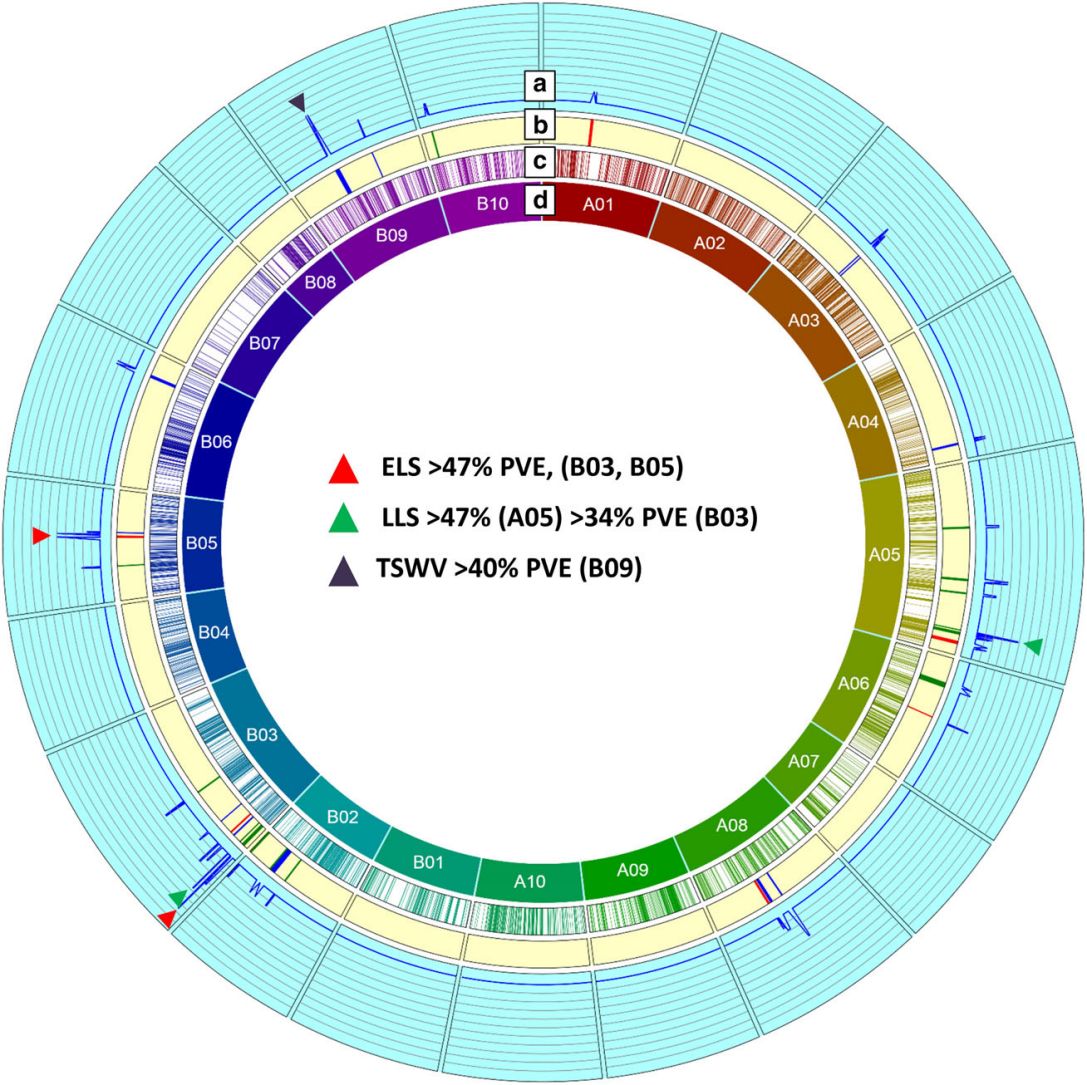
Genetic and QTL map of all the identified QTLs comprising SNP and SSR markers in the T‐population. (a) It represents the LOD values from 0 to 20 at an interval of 2 for each QTL, (b) it represents the QTLs identified in the study, (c) it represents the cM position of the SNP and SSR markers on the genetic map, and (d) it represents the LGs generated in the genetic map. Red corresponds to ELS QTL, green corresponds to LLS QTL, and black corresponds to TSWV QTL.

Quantitative trait locus mapping resulted in the identification of 35 QTLs with 6.3–47.6% PVE and LOD values between 2.5 and 11.3 associated with ELS, LLS and TSWV (Figures 2 and S5; Table 2). Of these 35 QTLs mapped onto 12 LGs, 31 were identified as major QTLs. Twenty QTLs were mapped onto six LGs of the A‐subgenome, and the remaining 15 QTLs were mapped onto six LGs of B‐subgenome. On the A‐subgenome, LG A06 harboured one minor QTL while the remaining 16 major QTLs were mapped on LGs A01, A03, A04, A05, A06 and A08. The B‐subgenome contained 15 major QTLs of the total 20 QTLs present on LGs B02, B03, B05, B06, B09 and B10.

**Table 2:** Details of QTLs identified for early leaf spot (ELS), late leaf spot (LLS) and TSWV resistance in the T‐population

S. No.	QTL	Year	Month	LG	Position (cM)	CI (cM)	Left Marker	Right Marker	LOD	PVE (%)	Additive effect (a0)	Physical distance of QTL (Mb)
Early Leaf Spot (ELS)												
1	qELS_T09_A08	2009	July	A08	102	100.7–102.9	A08_35596996	A08_35776787	3.02	12.42	0.56	0.17
2	qELS_T09_B05	2009	August	B05	80	78.0–80.2	B05_22527171	B05_20207815	10.02	47.42	−1.01	2.31
3	qELS_T11_A06	2011	August	A06	77	76.9–77.6	A06_14301316	A06_15094465	5.25	17.36	−0.27	0.79
4	qELS_T11_B03_1	2011	August	B03	29	27.7–29.2	A03_132207113	A03_132107871	5.96	20.35	0.28	0.099
5	qELS_T11_B03_2	2011	August	B03	12	11.8–12.5	A03_133651613	A03_133731756	11.35	47.38	−0.44	0.080
6	qELS_T13_A01	2013	July	A01	62	59.2–62.2	A05_ 99478314	A01_97507725	2.57	10.48	0.15	2.28^a^
7	qELS_T13_A05	2013	July	A05	225	221.7–225.3	B03_31318614	A05_7958319	3.04	14.14	−0.21	1.07^a^
Late Leaf Spot (LLS)												
8	qLLS_T13_B03_1	2013	August	B03	12	11.8–12.5	A03_133651613	A03_133731756	3.35	10.36	−0.16	0.080
9	qLLS_T11_B03_2	2011	September	B03	92	91.7–93.1	B03_121992379	B03_121040745	5.37	16.56	−0.21	0.95
10	qLLS_T11_B05	2011	September	B05	43	42.8–43.6	B05_41209982	B05_46042462	4.30	12.88	−0.18	4.83
11	qLLS_T11_A05_1	2011	September	A05	81	79.2–81.2	A05_95302689	A05_96201337	2.89	10.71	−0.19	0.89
12	qLLS_T11_A06	2011	September	A06	37	31.6–37.6	A06_8123560	A06_9957899	2.50	9.67	−0.18	1.83
13	qLLS_T11_B02_1	2011	September	B02	138	136.6–138.1	B02_105499048	B02_106618489	3.94	14.96	−0.22	1.11
14	qLLS_T12_A05_2	2012	September	A05	144	143.7 ‐146.1	A05_82270000	A05_54130650	5.46	20.65	0.40	28.13
15	qLLS_T12_A05_3	2012	September	A05	208	207.3–208.0	A05_20406182	B05_20992208	9.81	47.63	−0.62	1.85^a^
16	qLLS_T12_B02_2	2012	September	B02	82	81.5–82.9	A02_85035298	A02_85318601	3.44	12.88	−0.32	0.28
17	qLLS_T12_A05_4	2012	August	A05	161	160.8–162.4	A05_20504728	B09_138649848	2.92	10.27	−0.31	–
18	qLLS_T12_B02_3	2012	August	B02	106	98.6–106.5	B02_99031265	B03_14842626	3.29	11.78	−0.33	3.43^a^
19	qLLS_T12_B10	2012	August	B10	12	11.9–13.3	B10_10864883	B10_11224499	2.85	10.15	−0.31	0.35
20	qLLS_T12_A05_5	2012	September	A05	212	210.2–212.4	A05_15720064	A05_42599528	3.84	13.85	−0.30	26.87
21	qLLS_T13_A05_6	2013	August	A05	213	212.4–213.2	A05_10286363	A05_11746142	2.97	10.36	−0.27	1.45
22	qLLS_T13_B03_4	2013	August	B03	6	4.9–7.0	A03_134198144	A03_134634474	8.67	34.03	0.50	0.43
23	qLLS_T13_A05_5	2013	September	A05	212	210.2–212.4	A05_15720064	A05_42599528	2.83	12.24	−0.37	26.87
Tomato Spotted Wilt Virus (TSWV)												
24	qTSW_T10_A04	2010	July	A04	149	148.0–150.2	A04_1313025	A04_1109076	2.58	12.89	−0.38	0.2
25	qTSW_T10_A08_1	2010	July	A08	99	96.2–99.3	A08_35464654	A08_35813151	3.72	11.96	0.43	0.34
26	qTSW_T10_B03_1	2010	July	B03	47	46.3–47.0	A03_128864060	A03_128903550	2.62	8.10	−0.36	0.039
27	qTSW_T10_B09_1	2010	July	B09	110	109.6–110.3	A09_9631598	B09_14497666	4.33	14.10	−0.47	1.2^a^
28	qTSW_T10_B09_2	2010	July	B09	63	59.2–64.4	B09_6739506	B09_5189475	10.56	40.71	−0.80	1.55
29	qTSW_T10_A08_2	2010	August	A08	82	81.7–83.1	A08_29884265	A08_29203826	7.39	19.53	0.41	0.68
30	qTSW_T10_B02	2010	August	B02	103	98.6–104.3	B02_99031265	B02_101253445	2.61	6.32	0.23	2.22
31	qTSW_T10_B03_2	2010	August	B03	24	22.6–24.0	A03_131914876	A03_131407286	4.54	10.80	−0.30	0.5
32	qTSW_T10_B05	2010	August	B05	84	83.8–84.5	B05_19384851	A05_18990307	2.98	7.26	−0.25	0.14^a^
33	qTSW_T11_A03_1	2011	July	A03	75	74.5–75.2	A03_28765118	B03_31370666	5.30	16.78	0.34	2.6^a^
34	qTSW_T11_B06_1	2011	July	B06	142	140.0–143.2	B06_1837423	B06_1684497	4.57	14.40	−0.32	0.15
35	qTSW_T13_A03_2	2013	July	A03	78	77.9–78.6	A03_30749036	A03_30702259	3.66	14.46	0.32	0.046

a Physical distance calculated between the markers in case the two markers are not from the same chromosome. Nearest marker (closest to the QTL defining marker) of the same chromosome on that LG was used to calculate the physical distance. In cases, where even the nearest marker was from a different chromosome, physical distance was not calculated.

The QTLs were designated with initial letter *“q”* followed by the trait name, “T” year (T‐pop) and chromosome number. If there were more than one QTL for a trait in the same season, then it was suffixed by the numeric values as _1, _2 and so on.

For ELS, seven major QTLs were found on LGs A01, A03, A05, A06, A08, B03 and B05 with PVE ranging from 10.4% (*qELS_T13_A01)* to 47.4% (*qELS_T09_B05*). Five QTLs were present on the five LGs of A subgenome and the rest two on B03 and B05. Of all the seven major QTLs for ELS, *qELS_T09_B05* exhibited 47.4% PVE covering 2.3 Mb on the physical map. Two other QTLs, *qELS_T11_B03_2* and *qELS_T11_B03_1* mapped on B03 showed 47.3% and 20.35% PVE covered 80.13 Kb and 99.2 Kb, respectively, on the physical map. Both the QTLs with more than 47% PVE were contributed by the resistant parent Tifrunner. The QTL with 20.35% PVE (*qELS_T11_B03_1*) was contributed by GT‐C20 (Table 2).

Interestingly, SSRs flanking the LLS QTL were found to be within the SSRs marking the ELS QTL in the current genetic map of A05 (Figure S6). We identified 308 SNPs between the SSRs (TC40D04–GM1878) from an earlier report (Pandey *et al*., 2017b) on the current genetic map. Six of these 308 SNPs were found associated with three major QTLs for leaf spot on LG A05. First QTL (*qLLS_T12_A05_4*) was flanked by A05_20504728 and B09_138649848, which showed 10.27% PVE. The second major QTL identified was *qLLS_T12_A05_3* flanked by A05_20406182 and B05_20992208, which showed 47.8% PVE. The third QTL (*qLLS_T13_A05_6*) was flanked by A05_10286363 and A05_11746142 with 10.36% PVE. Seven of 15 QTLs including the one with maximum 47% PVE were found on LG A05, which is consistent with the earlier findings, where A05 contained the maximum number of QTLs (Pandey *et al*., 2017b).

In the case of TSWV, of the total, 13 QTLs were identified, and nine of these were major QTLs. These QTLs were present on LGs A03, A04, A08, B03, B06 and B09 ranging from PVE of 10.8% (*qTSW_T10_B09_2*) on B03 to 40.7% on B09 (Table 2). QTL on B03 with 10.8% PVE was flanked by SNPs identified from chromosome A03 (A03_131914876 and A03_131407286). Two common QTLs were identified between earlier study by Pandey *et al*. (2017b) and present study. These QTLs were *qTSW_T10_A04* and *qTSW_T10_B02* for July 2010 and August 2010. The other QTLs identified in the current study were novel.

### Environment interaction QTLs associated with disease resistance

A total of 13 QTL × E (environment) interactions were detected for three traits, of which nine QTLs were falling in the same genomic region, where major QTLs were also detected (Table S6). A total of three QTL × E interactions were detected for TSWV, and two of these were mapped on B09 with 3.42 and 8.35% PVE and another on A08 with 2.59%. In case of LLS, three QTL × E interactions were also identified out of which two were on A05 with 2.17%–4.82% and another on B03 with 5.95%. A total of seven QTL x E interactions were identified for ELS on B05, A08, B03, B09, A04, B04 and A08 with 11.89, 4.95, 3.75, 3.00, 2.82, 2.67 and 2.66% PVE, respectively.

### Epistatic QTLs associated with disease resistance

A total of 1048 epistatic QTL interactions were observed which included 268 for ELS, 505 for LLS and 275 for TSWV across the multiple environments. A maximum of 81 interactions for ELS_72_Tift_2013 and minimum of 28 interactions for LLS_93_Tift_2011 were identified. These interactions possessed both the positive and the negative additive effects on the traits. These epistatic interactions showed varied range of contribution towards the phenotypes as the PVE contributed by these detected environmental QTL (e‐QTL) were up to 59.43% PVE for ELS (ELS_8_Tift_2011), 44.91% PVE for LLS (LLS_91_Tift_2011) and 55.30% for TSWV (TSW_8_Tift_2010) (Table S7).

### Genomic region(s) and putative candidate genes associated with leaf spot and Tomato spotted wilt virus resistance

For ELS, two QTLs were identified with over 47% PVE on LG B03 (*qELS_T11_B03*) and B05 (*qELS_T11_B03*). The QTL mapped on LG B03 was flanked by markers A03_133651613 and A03_133731756, covering physical map distance of only 80.13 Kb. Of this 80 Kb region, only 10.5 Kb encompassed two genes and the remaining 69.5 Kb region was intergenic. The two genes in this region code for serine hydroxymethyl transferase (SHMT) and a rhodanese cell cycle control phosphatase superfamily protein. To scan the nearby regions, we looked for the genes 200 Kb upstream and downstream of the QTL. Flanking regions contained genes coding for xyloglucan endotransglucosylase/hydrolase, S‐adenosyl‐L‐homocysteine hydrolase and methyltransferase‐like protein, ATP/DNA‐binding protein, and small ubiquitin‐like modifier. A total of 15 SNPs were identified within the genes identified in these genes with a maximum of three SNPs each in two genes coding for UDP‐glycosyltransferase superfamily protein (*Aradu.VB4ZI*) and rhodanese/cell cycle control phosphatase superfamily protein (*Aradu.C56U2*) (Table S8). Other QTL on B05 was flanked by B05_22527171 and B05_20207815, encompassing >2.0 Mb distance on physical map. The 2.0 Mb QTL harboured 83 genes, including pectin esterase inhibitor, protein kinase, pentatricopeptide, NB‐ARC disease resistance, WRKY TF, F‐box/LRR‐repeat, MATE efflux family protein, cell wall protein‐like structure involved in cellulose microfibril organization, phosphotransferases and pathogenesis‐related genes as major defence‐related genes. The identified genes harboured 18 SNPs with three of these SNPs in gibberellin 2‐beta‐dioxygenase 8‐like protein‐coding gene (*Araip.A06C0*).

For LLS, a QTL on LG B03 flanked by A03_134198144 and A03_134634474 was reported with a PVE of 34%. It covered 0.43 Mb on the physical map encompassing a total of 29 genes including six unknown/uncharacterized protein‐coding genes. The other 23 genes code for proteins including a protein kinase family protein (leucine‐rich repeat‐containing N‐terminal), receptor‐like kinase (leucine‐rich repeat), WRKY TF, and heat‐shock transcription factor, glutathione S‐transferase, NADH:ubiquinone oxidoreductase intermediate‐associated protein, major intrinsic protein (MIP) family transporter (aquaporin like), zinc finger family protein, cinnamyl alcohol dehydrogenase, tetratricopeptide and transporter proteins. These genes are known to play an important role in plant defence against pathogen response including necrotrophic fungi. Genes within this QTL contained 10 SNPs. Another major QTL on LG A05 with 47% PVE covering 2 Mb harboured 78 genes coding for MATE efflux family protein, alanine and tryptophan aminotransferases, pathogenesis‐related protein, histone deacetylase, proteasome inhibitor, serine/threonine phosphatase, receptor kinase, protein kinase, pyruvate kinase, serine carboxypeptidase, ABC transporter family protein, xyloglucan xylosyltransferase and peptidase M50 family protein with 18 SNPs falling in the genes.

The major QTL for TSWV with more than 40% PVE on LG B09 was flanked by B09_5189475 and B09_6739506 and covered 1.55 Mb of physical distance, encompassing 114 genes including histone acyltransferase, heat‐shock protein, ATP‐binding ABC transporter, glutathione S‐transferase, cytochrome P450 superfamily protein, protein kinase superfamily protein, receptor‐like serine/threonine kinase, root hair defective 3 homolog 1‐like, MATE efflux, ethylene‐insensitive 3 family protein, disease resistance (TIR‐NBS‐LRR), glutamate dehydrogenase and others involved in direct or indirect defence responses against pathogens (Table S8). A total of 25 SNPs were identified within the genes in this QTL with a maximum of three effective SNPs in a putative candidate gene coding for protein kinase family protein (*Araip.RN7PY*).

### SNP efficiency and validation in RIL population

**Figure 3:**
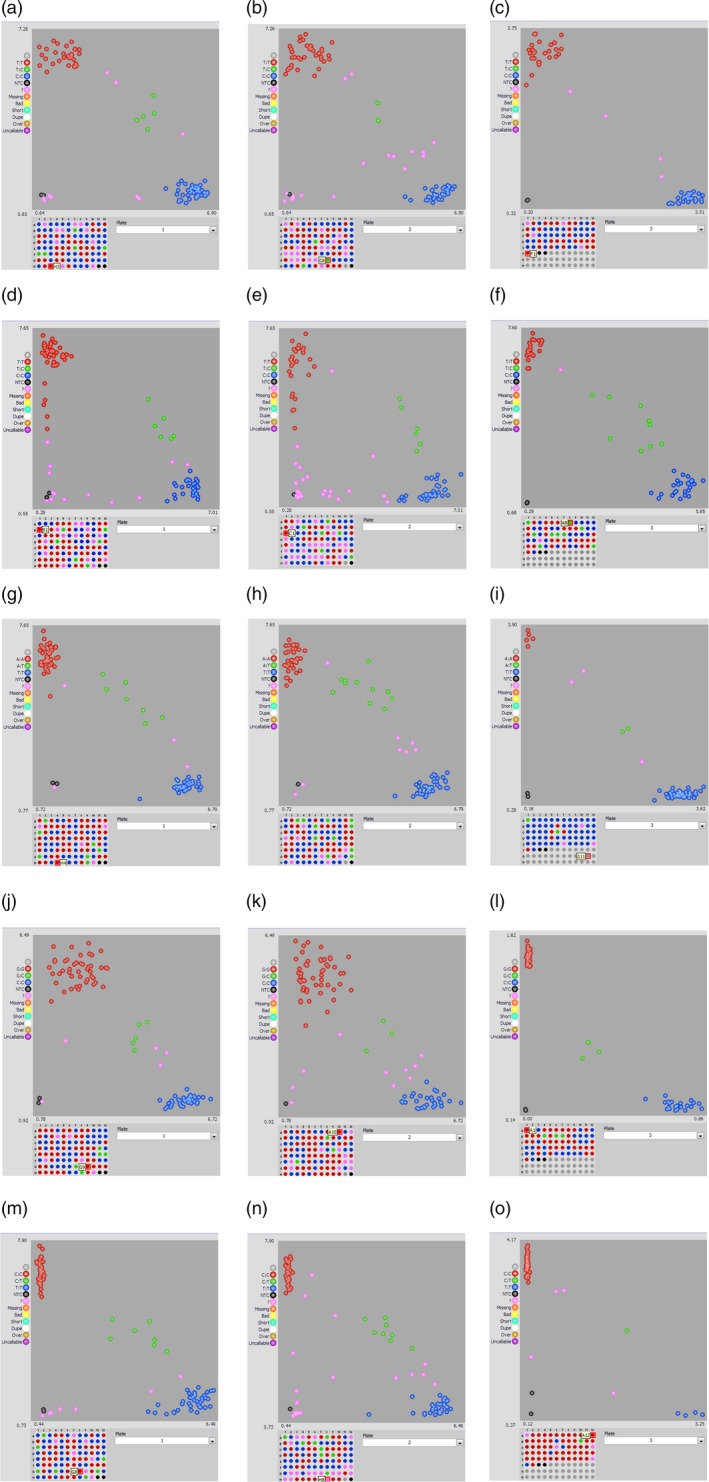
Snapshot displaying SNP genotyping with KASP assays using markers flanking the major QTLs for ELS and LLS. Different scenarios of validation of major QTL flanking SNPs B05_22527171 (a‐c) and A06_14301316 (d‐f) associated with ELS, A05_20406182 (g‐i), A03_134198144 (j‐l) and A05_82270000 (m‐o) associated with LLS segregating in the RIL population. Marker genotyping data generated for each genotype were viewed using the SNPviewer software (LGC Genomics). The scatter plot along *x* and *y* axes represents allelic discrimination for a particular marker in the examined population. Red and blue clusters represent the homozygous alleles showing polymorphism.

**Figure 4:**
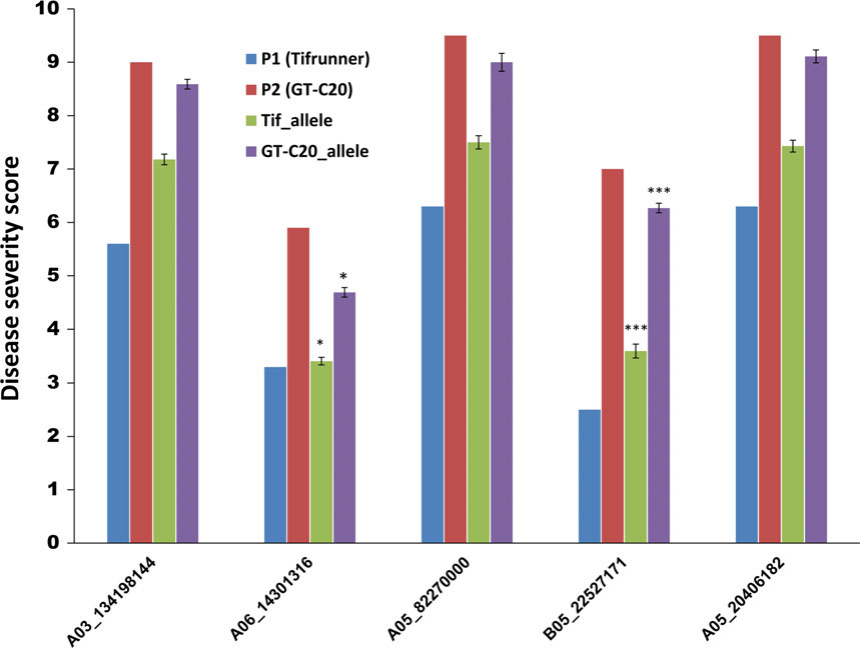
Graph showing correlation between the average disease severity scores (phenotype) and the five KASP validated SNPs. Vertical axis represents the average of disease severity, and horizontal axis shows the markers. Disease scores for the parents, Tifrunner (P1) and GT‐C20 (P2) for the season in that which QTL was detected are also shown for each marker. Unpaired *t*‐test was performed, and *P*‐values were estimated using the phenotyping data for number of alleles in population coming from Tifrunner and GT‐C20. Extremely statistically significant correlations are marked using *** with *P*‐value < 0.0001. Significant correlations are marked with *.

All SNPs present within the genes and in the flanking region of major QTLs were annotated for their effect and impact on gene function. SNPs within the coding region of a gene showed either a synonymous (does not change protein sequence) or a nonsynonymous (changes protein sequence) effect with moderate, high or low impact on gene function. Other SNPs were present either in the introns or untranslated regions (5′ or 3′) of a gene (Table S8). Five SNPs associated with ELS and LLS QTLs were also confirmed for their *in silico* genotype calls using KASP assay in the population. Overall, these SNPs showed efficiency of over 90%, implying that most of the *in silico* genotype calls were validated using the PCR‐based KASP assay (Table S9). KASP assay could clearly distinguish between the allelic variations in the population (Figure 3). To study correlation between the phenotype and KASP genotyping, we considered the extreme resistant and susceptible lines based on their phenotype ratings. One of the five markers associated with ELS (B05_22527171) showed not only good correlation with the *in silico* identified SNP calls, but also showed strong correlation with the phenotyping data of the RILs genotyped using this marker. Phenotype for the resistant lines carrying allele from the resistant parent (Tifrunner) genotyped using B05_22527171 averaged 3.5 and the susceptible lines carrying the alleles from susceptible parent (GT‐C20) averaged 6.2 (Figure 4). Other four markers (A03_134198144, A05_8227000, A05_20406182 and A06_14301316), however, did not show very strong correlation with the phenotyping ratings.

## Discussion

Evolution in sequencing and genotyping technologies has allowed for rapid advances in conducting genetic and breeding studies. Currently, these technologies have become not only cost‐effective and high throughput; both also provided opportunities to get deeper insights into the target genomic locations (Pandey *et al*., 2016). More and more deployment of these technologies has made available diagnostic markers for target traits for use in GAB towards accelerated crop improvement (Varshney *et al*., 2013). The recent origins of cultivated, allotetraploid peanut, a lack of optimal genetic resources and narrow genetic base has so far hampered the development of dense genetic maps for candidate gene identification. However, with the recent release of the reference genomes for both the diploid peanut progenitors, namely, *A. duranensis* (A) and *A. ipaensis* (B) (Bertioli *et al*., 2016), the application of NGS technologies in marker discovery and high‐density map generation is now possible for cultivated peanut. Therefore, here we used a WGRS strategy for genomewide SNP mining, genetic mapping and QTL analysis.

### Most dense genetic map for genetics and breeding applications

This WGRS method and subsequent analyses have already been used successfully in other legumes including chickpea (Kale *et al*., 2015) and soya bean (Qi *et al*., 2014; Xu *et al*., 2013). Using this approach, we have developed the densest genetic map currently available for cultivated peanut with 8,869 SNPs and 2156 mapped loci, which can be used for numerous applications including improvement of cultivated tetraploid peanut genome assembly (Peanutbase.org). While this genetic map has a high number of SNP markers (8869), only 24.3% (2156) of them represented the recombination as independent genetic loci. In comparison, other genetic maps such as that generated by Zhou *et al*. (2014) who used double‐digest restriction site‐associated DNA sequencing (ddRADseq) and successfully mapped 1621 SNPs and 64 SSRs, each representing an independent locus (1685 marker loci). The reduced loci:marker ratio may be due to the fact that these are haplotype‐based SNPs located in close physical proximity to each other on the respective chromosomes, which are tightly linked with little recombination between them. In addition, the reduced number of RIL individuals (91) used in the present study, compared to the 166 RILs used by Zhou *et al*. (2014), may have resulted in reduced mapping resolution to break apart the obtained haplotypes across multiple marker loci. Despite this, the density of the current genetic map demonstrates the power of this WGRS approach in peanut as the first report of its application in a biparental mapping population for trait mapping in this crop.

### B‐subgenome is lengthier but less diverse than A‐subgenome

More SNPs were identified and mapped on the A‐subgenome LGs compared to the B‐subgenome LGs despite the fact that B‐genome chromosomes are physically larger than A‐genome chromosomes. On this genetic map, a significant proportion of marker loci with assigned physical locations to a chromosome of one subgenome were mapped to respective homeologous positions on chromosomes of the other subgenome, indicating previous duplication events or mis‐assignments in these regions of genomes of diploid peanut that are the progenitors of cultivated tetraploid peanut. Most of this homeologous mapping of markers was seen between LGs A03 and B03, and the least were observed between A07, B07, and A08, B08 (Table S4, Figure 1a, b). Homeologous mapping has also been earlier reported in peanut between LGs A07 and B07, and A08 and B08 (Qin *et al*., 2012). This observation may also provide putative evidence for genetic exchanges occurring between the peanut subgenomes either during or following tetraploidization and the formation of cultivated peanut, a phenomenon also observed in earlier studies (Bertioli *et al*., 2016; Leal‐Bertioli *et al*., 2015). Also, a significant number of markers were placed on the genetic map on different chromosomes to which they were assigned. Most of these ‘translocated’ markers were seen between A06 and A10 and between A03 and A09, A10, fewer were seen on B04 (Table 1, Figure 1a, b). These ‘translocated’ markers observed might be due to reciprocal translocation occurring because of the exchange of two terminal segments between two nonhomologous chromosomes (Farré *et al*., 2011). As a result, recombination between loci around translocation breakpoints is suppressed leading to linkage between markers in these regions, which explains the linkage detected between markers lying on different chromosomes seen in the current study. Such translocations are well documented in various crops including barley (Farré *et al*., 2011), soya bean (Mahama and Palmer, 2003), *Prunus spp*. (Jáuregui *et al*., 2001), lentils (Tadmor *et al*., 1987), pea (Kosterin *et al*., 1999) and peanut (Qin *et al*., 2012). Some of these observed ‘translocated’ markers might be also due to artefacts and mis‐assignments because of the highly repetitive structure of the genome (Bertioli *et al*., 2016).

### QTLs and potential candidate genes related to defense against leaf spots and TSWV

Disease resistance is a highly heritable trait of great value to crop production systems. Efforts have been made to identify the QTLs/candidate genes for important diseases such as ELS, LLS and TSWV in peanut (Khera *et al*., 2016; Pandey *et al*., 2017a,2017c). In the present study, 20 QTLs were identified on A‐subgenome LGs and 15 on B‐subgenome LGs. Of the 35 identified QTLs, 24 (65%) were flanked by markers from A‐subgenome. In contrast, Bertioli *et al*. (2016) identified 345 and 397 NB‐LRR (nucleotide‐binding‐leucine‐rich repeat) genes in the A and the B genomes sequenced, respectively.

In earlier studies with sparse genetic maps and few polymorphic markers, the maximum PVE of detected QTL was 27.35% (Pandey *et al*., 2017a; Wang *et al*., 2013) and up to 29.14% in another RIL population, SunOleic97R × NC 94022 (S‐population) (Khera *et al*., 2016). All these studies resulted in broad QTL regions with no candidate gene identification being possible. The current study identified seven major QTLs for ELS with four of them having negative additive effects suggesting contribution by parent 1, Tifrunner (R), of which two were located on LGs B03 and B05 contributing over 47% PVE each (Table 2, Figure 2). In an earlier study, LG A03 was reported to harbour major ELS resistance‐related QTL (Pandey *et al*., 2017a). The two major QTLs for ELS on LG B03 were flanked by homeologous markers from chromosome A03, suggesting that these regions of chromosome A03 may constitute a hot spot for genes responsible for ELS resistance. In a recent study using QTL‐seq approach in a TAG24 × GPBD4 RIL population, a 2.98 Mb (131.67–134.65 Mb) genomic region on chromosome A03, which overlaps with the region indicated in the present study to contain these two major ELS‐resistant QTLs (132.10–132.20 Mb and 133.65–133.73 Mb, respectively), was also found to contribute to LLS resistance (Pandey *et al*., 2017c). This supports the notion that this region on chromosome A03 has genes playing role in defence against leaf spots. However, it is also possible that although ELS and LLS classification was based on observations of predominant signs and symptoms in the fields used for disease phenotyping, both ELS and LLS likely co‐occurred in the field, and the detected QTL may not be specific. Greenhouse‐based phenotyping with controlled inoculations will be required to confirm this conclusion.

Major QTL regions on LG B05 for ELS, and A05 and B03 for ELS and LLS also contained leucine‐rich repeat (LRR), NB‐ARC, or receptor kinase genes which may function as putative R‐genes. Interestingly, while the major QTL region found for ELS was also observed for LLS, but at a lesser PVE (qELS_T11_B03_2 and qLLS_T13_B03_1), there was another major QTL for LLS on B03 associated with different markers which contained a putative R‐gene. This may indicate a degree of R‐gene specificity for each pathogen based on the observed PVEs associated with each QTL. The association of markers from A05 and A03 based on diploid reference sequences with major LLS QTL on LGs A05 and B03, respectively, while the ELS R‐gene containing QTL on LG B05 is associated with markers from chromosome B05 in the diploid reference genome on the current map may also indicate subgenome‐associated specificity. Given the possibility, however, of co‐occurrence of both pathogens during the disease ratings, further studies with controlled inoculation will be required to explore this hypothesis more completely.

In addition to R‐genes, genes coding for serine hydroxymethyltransferase (SHMT), rhodanese cell cycle control phosphatase superfamily protein and ubiquitin‐protein ligase were identified in the region harboured by above‐mentioned two major QTLs on LG B03. It has been reported that mutation of *shmt1* compromises host resistance to biotrophic and necrotrophic foliar pathogens (Moreno *et al*., 2005). Rhodanese is a detoxifying enzyme known to detoxify the harmful effects of HCN produced in plants as an antifungal agent in response to phytopathogenic fungi (Miller and Conn, 1980; Osbourn, 1996), whereas ubiquitin‐protein ligase is known to be involved in the initial steps of pathogen perception and in the regulation of downstream defence signalling (Duplan and Rivas, 2014).

For LLS, QTLs with only 15%–17% PVE were earlier identified on LGs A05, A06, A07 and B03, B05 using T‐ and S‐populations, respectively (Khera *et al*., 2016; Pandey *et al*., 2017a) when compared to 17%, 34% and 47.6% QTLs in current study. Genes identified in these QTLs like glutathione S‐transferase are known to be involved in resistance responses to leaf spot disease caused by *Cercosporidium personatum* (Luo *et al*., 2004). Other genes such as heat shock, MYB, MIP (aquaporins) TFs and receptor kinase have been known play a significant role in pathogen response (Katiyar *et al*., 2012; Kumar *et al*., 2009; de Paula Santos Martins *et al*., 2015; Tang *et al*., 2017).

Finally, for TSWV, previous studies had identified QTLs with PVE of 5.2‐29.14% in T‐ and S‐populations (Khera *et al*., 2016; Pandey *et al*., 2017b; Qin *et al*., 2012; Wang *et al*., 2013) in comparison with nine major QTLs were identified with PVE from 10.8% to 40.71% in the current study. QTL with maximum PVE was dissected to look for the genes lying in it. Mostly, genes coding for LRR disease resistance protein, protein and receptor kinases, glutamate dehydrogenase (GDH), serine acyltransferase and root hair defective 3 homolog 1‐like were identified. GDH adds an amino group to 2‐oxoglutarate, to generate glutamate as a TCA intermediate. This function of the enzyme has been reported to occur in response to bacterial as well as viral invasions (Pageau *et al*., 2006). Cysteine synthesis in plants is carried out by serine acetyltransferase. Cysteine acts as a reduced sulphur donor molecule involved in the synthesis of essential biomolecules and defence compounds (Romero *et al*., 2014). Root hair defective 3 homolog 1‐like is a GTP‐binding protein involved in cell wall expansion (Wang *et al*., 1997).

### Phenotypic variance of disease resistance traits affected by epistatic and QTL x E interactions

Identification of QTL with additive, stable across different environments and epistasis is very crucial in molecular breeding programmes. Of the several interactions, nine QTL × E interactions were identified on the same regions, where major QTLs were detected, for example three major QTLs for TSWV (*qTSW_T10_B09_2*,* qTSW_T10_B09_1*,* qTSW_T10_A08_1*) with up to 40.71% PVE. Similarly, four major QTLs (*qLLS_T13_B03_1, qLLS_T13_B03_3, qLLS_T12_A05_2, qLLS_T12_A05_3*) with up to 47.63% for LLS, while three major QTLs (*qELS_T09_B05, qELS_T09_A08, qELS_T11_B03_2*) with up to 47.42% for ELS. Further, the epistatic QTL study detected hundreds of QTL–QTL interactions across the genome for ELS, LLS and TSW. Two SNP loci (A05_20406177, A05_82270000) associated with LLS and B05_22527171 associated with ELS were successfully validated with KASP assay. These QTL–QTL interactions indirectly show the cross‐talk of the homeologous genes across the A and B subgenome. This detailed study of QTL × E and QTL × QTL along with the major QTL showed the importance of the genomic interaction and suggest to introgress favourable genomic loci including main effect, epistasis and QTL × Environment interactions for achieving desired phenotype through the molecular breeding programme.

### Validated diagnostic markers available for use in molecular breeding

Robust KASP genotyping assays were also developed for ELS‐ and LLS‐related disease traits using the SNP markers. It remarkably demonstrated the robustness and accuracy of KASP markers as these markers were validated in RIL population in correlation with *in silico* genotype calls. Studies in other legumes like pea, pigeon pea, chickpea, and soya bean using KASP markers have shown promising results (Boutet *et al*., 2016; Hiremath *et al*., 2012; Patil *et al*., 2017; Saxena *et al*., 2014). Studies in chickpea, pigeon pea and peanut identified 66.8%, 75.86% and 80% of the KASP markers to be polymorphic (Saxena *et al*., 2014). Also, in the current study, a very high level of consistency (~90%) between the *in silico* called SNPs and KASP validated data was reported. While significant, though numerically limited separation between resistant and susceptible genotyped plants when considering average phenotypic disease ratings could be observed for single markers, utilizing multiple major QTL‐associated KASP markers will provide a means of improved predictive selection. These markers can be implemented in peanut breeding programmes, which can augment GAB of disease‐related traits with greater efficiency and accuracy. The validated KASP markers can also be used for allele mining, marker‐assisted selection and forward breeding programmes. Further studies will be conducted to implement these markers as diagnostic markers in other resistant and susceptible genetic backgrounds.

## Conclusions

The development of high‐density genetic maps is a requirement for the fine mapping of loci contributing to quantitative traits such as disease resistance. Here, we have presented the densest genetic map currently available for cultivated peanut and the first to be generated using a WGRS approach in peanut. With this high‐density map, 35 major QTLs were identified for important diseases of peanut including ELS (47.4% PVE), LLS (47.6% PVE) and TSWV (40.7% PVE). This also allowed for the development of KASP markers for SNPs associated with ELS and LLS QTLs. These PCR‐based markers provide peanut breeders a useful tool to improve marker‐assisted selection in breeding programmes. In addition, the generation of this high‐density genetic map also allows for the correction of future genome assemblies for tetraploid peanut.

## Materials and methods

### Plant materials and generation of phenotyping data

Two peanut inbred lines, Tifrunner and GT‐C20, were selected as parents to develop a RIL mapping population. Tifrunner, the female parent (runner market type) has a high level of resistance to TSWV, moderate resistance to ELS and LLS, and late maturity (Holbrook and Culbreath, 2007). GT‐C20, the male parent (Spanish market type), is susceptible to all three diseases (Liang *et al*., 2005). This RIL population has been used in genetic map construction and QTL analysis (Pandey *et al*., 2014, 2017a; Qin *et al*., 2012; Wang *et al*., 2012), primarily using SSR markers. For this study, a subset of 91 RILs were selected for whole‐genome resequencing.

The whole RIL population was phenotyped in field for ELS, LLS and TSWV disease severity from 2009 to 2013. There were two plantings for 2011, 2012 and 2013, an early planting in April and a late planting in May in each year. Overall, there were a total of eight plantings each with three replications using a randomized complete block design. Disease severity ratings for the three diseases (ELS, LLS and TSWV) were performed at three different dates in growing seasons in July, August and September. Ratings for ELS and LLS were measured using the Florida 1–10 scale (Chiteka *et al*., 1988). TSWV disease severity was measured on a 1–10 disease severity in plots exhibiting typical symptoms such as stunting, ringspot, leaf necrosis and chlorosis (Baldessari, 2008; Culbreath *et al*., 2003).

### Library construction and sequencing

Young leaf tissues from each of the 91 selected RILs along with the parental lines were used for genomic DNA isolation using the CTAB method and quantified as described by Wang *et al*. (2016). A whole‐genome shotgun sequencing strategy was then used to construct the paired‐end libraries. Paired‐end sequencing libraries were sequenced with read length of 100 bp using an Illumina HiSeq 2000 platform (Illumina, San Diego, CA). Parental genotypes were sequenced separately at a high sequencing depth, ~100× for Tifrunner with insert size ranging from 170 bp to 40 kb, and ~10× for GT‐C20 with an insert size of 500 bp. Individual RILs were sequenced at ~2–5× coverage. Filtered reads were used for alignment to the reference genome assemblies of *A. duranensis* (v1, peanutbase.org) and *A. ipaensis* (v1, peanutbase.org) separately and used for SNP identification and genotyping. The binary alignment mapping (BAM) files using the read data have been submitted to the SRA database at NCBI under the SRA accession: SRP134206.

### Sequence analysis and SNP discovery for genetic analysis

Filtered reads from Tifrunner, GT‐C20 and the individual RILs (fastq formatted) were mapped to the genome assemblies of *A. duranensis* (v1, peanutbase.org) and *A. ipaensis* (v1, peanutbase.org) separately using the Burrows–Wheeler alignment (BWA) tool with default parameters (Li and Durbin, 2009). Alignment files were used to identify polymorphic haplotypes using a novel pipeline (Clevenger *et al*., unpublished), which is an improved version of SWEEP described by Clevenger and Ozias‐Akins (2015). The haplotypes were generated within a read, that is less than 100 bp. Briefly, all potential polymorphisms were called using Samtools mpileup. A sliding window strategy was used to visit every two base haplotype of potential polymorphisms that were physically within 100 bp. At each haplotype locus, every distinct haplotype along with observed counts for each haplotype within each genotype was collected. A polymorphic haplotype was identified when meeting the following criteria: (i) All haplotypes for both genotypes were observed more than once, (ii) at least one haplotype differed between the genotypes at one base while the other base remained constant and (iii) the haplotype with the least number of observations had within 25% of the observations of the haplotype with the most observations. Identified putative polymorphic haplotype SNPs were then used to genotype the population. Each individual was mapped to the *A. ipaensis* and *A. duranensis* genome separately. For each individual, the A‐genome‐derived haplotypes were scored in the alignment mapped to *A. duranensis* and the B‐genome‐derived haplotypes were scored in the alignment mapped to *A. ipaensis*. At each potential polymorphic locus, if the polymorphic haplotype from one parent is observed more than once and the haplotype from the other parent is not observed, the individual was scored to have the allele from the parent that the observed haplotype is derived. If both haplotypes were observed or one haplotype was only observed one time, the individual was scored as missing data.

### Linkage map construction, identification of homeologous and translocated markers

Filtered SNPs with less than 20% missing data were used to construct the genetic maps using QTL IciMapping v4.1 (Meng *et al*., 2015). The chi‐square (χ^2^) values calculated for each SNP marker were used to determine the goodness of fit to the expected 1:1 segregation ratio. Highly distorted and unlinked markers were not considered for the linkage map construction. The markers were grouped at LOD ≥ 4 and ordered using the nnTwoOpt algorithm. Kosambi's mapping function was used for converting the recombination frequency into map distance in centiMorgan (cM). Homeologous SNPs are defined as the ones that were identified from A‐genome chromosome of the reference diploid genome, but mapped on the corresponding B‐genome LG. For example, when a SNP called on chromosome A01 was mapped to LG B01 or a SNP called on chromosome B01 was mapped to LG A01, they were regarded as homeologous. Translocated SNPs are defined as the ones that are identified on A‐genome chromosome of the reference diploid genome; however, on the linkage map, the same SNPs were mapped on any A‐ or B‐genome linkage groups except the corresponding B‐genome LG. For example, SNPs called on chromosome A01 when mapped to any of the A‐ or B‐LG except A01 and B01 were regarded as translocated. Circa (http://omgenomics.com/circa/) was used to plot circos to demonstrate the homeologous and translocated markers.

### Epistatic and environmental QTL analysis

The genotyping and phenotyping data of population were used for QTL analysis using the inclusive composite interval mapping (ICIM) function of QTL IciMapping v4.1. A QTL was considered to be major only if had a LOD ≥ 3 and PVE explained >10%. The genetic map information together with phenotyping data was used for the identification of epistatic and environmental QTL interaction studies for ELS, LLS and TSWV using ICIM mapping. BIP (biparental) and MET (QTL by environment) functionalities of inclusive composite interval mapping (ICIM) was used for identification of epistasis and environmental QTLs, respectively. The additive (two‐dimensional scanning, ICIM‐EPI) method with 5 cM step and 0.001 probability mapping parameters in stepwise regression were employed in QTL analysis. For environmental QTL analysis, one input file for each in multiple environments was used. Also, the largest *P*‐value for removing variables was assumed to be two times the value of P‐value for entering variables in stepwise regression. The missing phenotypic data were replaced by the phenotypic mean of the trait built‐in function.

### KASP assay development

The KASP genotyping assay is a fluorescence‐based assay for identification of biallelic SNPs. Two allele‐specific forward primers along with tail sequences and one common reverse primer were synthesized (Table S10). The reaction mixture was prepared following the manufacturer's instructions (KBioscience; http://www.lgcgroup.com/products/kasp-genotyping-chemistry/#.VsZK7PkrKM8). KASP reaction mixture with 10 μL final reaction volume contained 5 μL KASP master mix, 0.14 μL primer mix, 2 μL of 10–20 ng/μL genomic DNA and 2.86 μL of water. PCR conditions used were as follows: 15 min at 95°C followed by 10 touchdown cycles of 20 s at 94°C and 1 min at 61–55°C (dropping 0.6°C per cycle), and then 26 cycles of 20 s at 94°C and 1 min at 55°C. KASP marker data were then analysed using SNPviewer software (LGC Genomics) (http://www.lgcgroup.com) to generate genotype calls for each RIL and parental line, and were correlated with observed disease ratings.

## Author contributions

GA performed most of the experiments, conducted sequence alignment, SNP calling and drafted the manuscript. JC developed the programme, conducted sequence analysis and called the SNPs. MKP contributed in planning and designing the experiment, data analysis and manuscript revision. HW and YC prepared the DNA. HW, CCH and AKC performed field phenotyping. HW, JCF, YS and DC participated the mapping and designed primers. MKP and YS did the environment and QTL interaction studies. XL and GH generated the sequence data. RD helped in statistical analyses. XW, DJB, SAJ and PO provided inputs and discussion. BG conceived the plan; BG and RKV designed and finalized the manuscript.

## Conflict of interest statement

The authors declare that there is no conflict of interests.

## Supporting information


**Figure S1** Phenotypic distribution of ELS, LLS and TSWV in T‐pop RILs during different seasons.

**Figure S2** Percentage reads mapped to the diploid reference A‐ and B‐genome in each RIL and the two parents.

**Figure S3** Frequency histogram of the percentage of missing data points in WGRS of 91 RILs with 16 674 polymorphic SNPs in the population.

**Figure S4** Distribution of markers on linkage groups.

**Figure S5** QTL maps showing the major QTL peaks at different LODs on vertical axis.

**Figure S6** Genetic and QTL map of major QTLs (>10% PVE) comprising SNP and SSR markers in Tifrunner × GT‐C20 population in peanut (Pandey *et al*., 2017a).

**Table S1** Phenotypic variation of diseases (ELS, LLS and TSWV) in T‐population parents and RILs.
**Table S2** Overview of the WGRS data and alignment to the reference genome.
**Table S3** Summary of SNPs detected between Tifrunner and GT‐C20 and SNPs used in RIL population.
**Table S4** Number of homeologus and translocated markers.
**Table S5** Effect of major QTL associated SSR markers reported in earlier study on the QTL and length of current genetic map.
**Table S6** Summary of environment QTLs detected in T‐population.
**Table S7** Summary of the epistatic QTLs detected across different environments and locations for the ELS, LLS and TSWV in the RILs.
**Table S8** SNPs in putative candidate genes underlying the major QTLs for ELS, LLS and TSWV resistance with their effect and impact.
**Table S9** SNP efficiency of markers validated using KASP assay.
**Table S10** List of primer sequences for KASP assay of SNPs developed and validated for Early Leaf Spot (ELS) and Late Leaf Spot (LLS).

## References

[pbi12930-bib-0001] Baldessari, J. J. (2008) Genetics of Tomato Spotted Wilt Virus Resistance in Peanut (Arachis hypogaea L.). Gainesville, FL: University of Florida.

[pbi12930-bib-0002] Bertioli, D.J. , Cannon, S.B. , Froenicke, L. , Huang, G. , Farmer, A.D. , Ethalinda, K.S.C. , Xin, L. *et al* (2016) The genome sequences of *Arachis duranensis* and *Arachis ipaensis*, the diploid ancestors of cultivated peanut. Nat. Genet. 48, 438–446. 26901068 10.1038/ng.3517

[pbi12930-bib-0003] Boutet, G. , Carvalho, S.A. , Falque, M. , Peterlongo, P. , Lhuillier, E. , Bouchez, O. , Lavaud, C. *et al* (2016) SNP discovery and genetic mapping using genotyping by sequencing of whole genome genomic DNA from a pea RIL population. BMC Genom. 17, 121. 10.1186/s12864-016-2447-2 PMC4758021 26892170

[pbi12930-bib-0004] Chen, X. , Li, H. , Pandey, M.K. , Yang, Q. , Wang, X. , Garg, V. , Li, H. *et al* (2016) Draft genome of the peanut A‐genome progenitor (*Arachis duranensis*) provides insights into geocarpy, oil biosynthesis and allergens. Proc. Natl Acad. Sci. 113, 6785–6790. 27247390 10.1073/pnas.1600899113 PMC4914189

[pbi12930-bib-0005] Chiteka, Z.A. , Gorbet, D.W. , Knauft, D.A. , Shokes, F.M. and Kucharek, T.A. (1988) Components of resistance to late leaf spot in peanut. II. Correlations among components and their significance in breeding for resistance. Peanut Sci. 15, 76–81.

[pbi12930-bib-0006] Clevenger, J.P. and Ozias‐Akins, P. (2015) SWEEP: A tool for filtering high‐quality SNPs in polyploid crops. G3: Genes Genom., Genet. 5, 1797–1803. 10.1534/g3.115.019703 PMC4555216 26153076

[pbi12930-bib-0007] Clevenger, J. , Chu, Y. , Chavarro, C. , Agarwal, G. , Bertioli, D.J. , Leal‐Bertioli, S.C. , Pandey, M.K. *et al* (2017) Genome‐wide SNP genotyping resolves signatures of selection and tetrasomic recombination in peanut. Mol. Plant. 10, 309–322. 27993622 10.1016/j.molp.2016.11.015 PMC5315502

[pbi12930-bib-0008] Culbreath, A.K. , Todd, J.W. and Brown, S.L. (2003) Epidemiology and management of tomato spotted wilt in peanut. Annu. Rev. Phytopathol. 41, 53–75. 12704217 10.1146/annurev.phyto.41.052002.095522

[pbi12930-bib-0009] Duplan, V. and Rivas, S. (2014) E3 ubiquitin‐ligases and their target proteins during the regulation of plant innate immunity. Front. Plant Sci. 5, 42. 24592270 10.3389/fpls.2014.00042 PMC3923142

[pbi12930-bib-0010] Farré, A. , Benito, I.L. , Cistué, L. , De Jong, J.H. , Romagosa, I. and Jansen, J. (2011) Linkage map construction involving a reciprocal translocation. Theor. Appl. Genet. 122, 1029–1037. 21153624 10.1007/s00122-010-1507-2 PMC3043263

[pbi12930-bib-0011] Food and Agriculture Organization (FAO) . The State of Food and Agricultural Biotechnology, Meeting the Needs of the Poor. Rome, Italy: Food and Agricultural Organization of the United Nations 2004.

[pbi12930-bib-0012] Guo, B.Z. , Pandey, M.K. , He, G. , Zhang, X.Y. , Liao, B. , Culbreath, A.K. , Varshney, R.K. *et al* (2013) Recent advances in molecular genetic linkage maps of cultivated peanut (*Arachis hypogaea* L.). Peanut Sci. 40, 95–106.

[pbi12930-bib-0013] Halward, T. , Stalker, T. , LaRue, E. and Kochert, G. (1992) Use of single‐primer DNA amplifications in genetic studies of peanut (*Arachis hypogaea* L.). Plant Mol. Biol. 18, 315–325. 1731991 10.1007/BF00034958

[pbi12930-bib-0014] Hiremath, P.J. , Kumar, A. , Penmetsa, R.V. , Farmer, A. , Schlueter, J.A. , Chamarthi, S.K. , Whaley, A.M. *et al* (2012) Large‐scale development of cost‐effective SNP marker assays for diversity assessment and genetic mapping in chickpea and comparative mapping in legumes. Plant Biotechnol. J. 10, 716–732. 22703242 10.1111/j.1467-7652.2012.00710.x PMC3465799

[pbi12930-bib-0015] Holbrook, C.C. and Culbreath, A.K. (2007) Registration of ‘Tifrunner’ peanut. J. Plant Regist. 1, 10–3198.

[pbi12930-bib-0016] Jáuregui, B. , De Vicente, M.C. , Messeguer, R. , Felipe, A. , Bonnet, A. , Salesses, G. and Arús, P. (2001) A reciprocal translocation between ‘Garfi’ almond and ‘Nemared’ peach. Theor. Appl. Genet. 102, 1169–1176.

[pbi12930-bib-0017] Kale, S.M. , Jaganathan, D. , Ruperao, P. , Chen, C. , Punna, R. , Kudapa, H. , Thudi, M. *et al* (2015) Prioritization of candidate genes in “QTL‐hotspot” region for drought tolerance in chickpea (*Cicer arietinum* L.). Sci. Rep. 5, 15296. 26478518 10.1038/srep15296 PMC4609953

[pbi12930-bib-0018] Katiyar, A. , Smita, S. , Lenka, S.K. , Rajwanshi, R. , Chinnusamy, V. and Bansal, K.C. (2012) Genome‐wide classification and expression analysis of MYB transcription factor families in rice and Arabidopsis. BMC Genom. 13, 544. 10.1186/1471-2164-13-544 PMC3542171 23050870

[pbi12930-bib-0019] Khera, P. , Pandey, M.K. , Wang, H. , Feng, S. , Qiao, L. , Culbreath, A.K. , Kale, S. *et al* (2016) Mapping quantitative trait loci of resistance to tomato spotted wilt virus and leaf spots in a recombinant inbred line population of peanut (*Arachis hypogaea* L.) from SunOleic 97R and NC94022. PLoS ONE, 11, e0158452. 27427980 10.1371/journal.pone.0158452 PMC4948827

[pbi12930-bib-0020] Kosterin, O. , Pukhnachev, N. , Gorel, F. and Berdnikov, V. (1999) Location of the breakpoints of four reciprocal translocations involving group V and their influence on recombination distances between neighbouring markers. Pisum Genet. 31, 13–20.

[pbi12930-bib-0021] Kumar, M. , Busch, W. , Birke, H. , Kemmerling, B. , Nürnberger, T. and Schöffl, F. (2009) Heat shock factors HsfB1 and HsfB2b are involved in the regulation of Pdf1.2 expression and pathogen resistance in *Arabidopsis* . Mol. Plant. 2, 152–165. 19529832 10.1093/mp/ssn095 PMC2639743

[pbi12930-bib-0022] Leal‐Bertioli, S. , Shirasawa, K. , Abernathy, B. , Moretzsohn, M. , Chavarro, C. , Clevenger, J. , Ozias‐Akins, P. *et al* (2015) Tetrasomic recombination is surprisingly frequent in allotetraploid Arachis. Genetics, 199, 1093–1105. 25701284 10.1534/genetics.115.174607 PMC4391553

[pbi12930-bib-0023] Li, H. and Durbin, R. (2009) Fast and accurate short read alignment with Burrows‐Wheeler transform. Bioinformatics, 25, 1754–1760. 19451168 10.1093/bioinformatics/btp324 PMC2705234

[pbi12930-bib-0024] Liang, X. , Holbrook, C.C. , Lynch, R.E. and Guo, B.Z. (2005) Beta‐1,3‐glucanase activity in peanut seed (*Arachis hypogaea*) is induced by inoculation with Aspergillus flavus and copurifies with a conglutin‐like protein. Phytopathology, 95, 506–511. 18943315 10.1094/PHYTO-95-0506

[pbi12930-bib-0025] Luo, H. , Dang, P. , Bausher, M.G. , Holbrook, C.C. , Lee, R.D. , Lynch, R.E. and Guo, B.Z. (2004) Identification of transcripts involved in resistance responses to leaf spot disease caused by *Cercosporidium personatum* in Peanut (*Arachis hypogaea*). Phytopathology, 95, 381–387. 10.1094/PHYTO-95-0381 18943040

[pbi12930-bib-0026] Mahama, A.A. and Palmer, R.G. (2003) Translocation breakpoints in soybean classical genetic linkage groups 6 and 8. Crop Sci. 43, 1602–1609.

[pbi12930-bib-0027] Meng, L. , Li, H. , Zhang, L. and Wang, J. (2015) QTL IciMapping: Integrated software for genetic linkage map construction and quantitative trait locus mapping in biparental populations. Crop J. 3, 269–283.

[pbi12930-bib-0028] Miller, J.M. and Conn, E.E. (1980) Metabolism of hydrogen cyanide by higher plants. Plant Physiol. 65, 1199–1202. 16661359 10.1104/pp.65.6.1199 PMC440509

[pbi12930-bib-0029] Moreno, J.I. , Martín, R. and Castresana, C. (2005) Arabidopsis SHMT1, a serine hydroxymethyltransferase that functions in the photorespiratory pathway influences resistance to biotic and abiotic stress. Plant J. 41, 451–463. 15659103 10.1111/j.1365-313X.2004.02311.x

[pbi12930-bib-0030] Moretzsohn, M.C. , Gouvea, E.G. , Inglis, P.W. , Leal‐Bertioli, S.C.M. , Valls, J.F.M. and Bertioli, D.J. (2013) A study of the relationships of cultivated peanut (*Arachis hypogaea*) and its most closely related wild species using intron sequences and microsatellite markers. Ann. Bot. 111, 113–126. 23131301 10.1093/aob/mcs237 PMC3523650

[pbi12930-bib-0031] Nayak, S.N. , Agarwal, G. , Pandey, M.K. , Sudini, H.K. , Jayale, A.S. , Purohit, S. , Desai, A. *et al* (2017) *Aspergillus flavus* infection triggered immune responses and host‐pathogen cross‐talks in groundnut during in‐vitro seed colonization. Sci. Rep. 7, 9659. 28851929 10.1038/s41598-017-09260-8 PMC5574979

[pbi12930-bib-0032] Ogwulumba, S.I. , Ugwuoke, K.I. and Iloba, C. (2008) Prophylactic effect of paw‐paw leaf and bitter leaf extracts on the incidence of foliar myco‐pathogens of groundnut (*Arachis hypogaea* L.) in Ishiagu, Nigeria. Afr. J. Biotechnol. 7, 2878–2880.

[pbi12930-bib-0033] Osbourn, A.E. (1996) Preformed antimicrobial compounds and plant defense against fungal attack. Plant Cell, 8, 1821–1831. 12239364 10.1105/tpc.8.10.1821 PMC161317

[pbi12930-bib-0034] Pageau, K. , Reisdorf‐Cren, M. , Morot‐Gaudry, J.F. and Masclaux‐Daubresse, C. (2006) The two senescence‐related markers, GS1 (cytosolic glutamine synthetase) and GDH (glutamate dehydrogenase), involved in nitrogen mobilization, are differentially regulated during pathogen attack and by stress hormones and reactive oxygen species in *Nicotiana tabacum* L. leaves. J. Exp. Bot. 57, 547–557. 16377736 10.1093/jxb/erj035

[pbi12930-bib-0035] Pandey, M.K. , Monyo, E. , Ozias‐Akins, P. , Liang, X. , Guimarães, P. , Nigam, S.N. , Upadhyaya, H.D. *et al* (2012) Advances in Arachis genomics for peanut improvement. Biotechnol. Adv. 30, 639–651. 22094114 10.1016/j.biotechadv.2011.11.001

[pbi12930-bib-0036] Pandey, M.K. , Wang, M.L. , Qiao, L. , Feng, S. , Khera, P. , Wang, H. , Tonnis, B. *et al* (2014) Identification of QTLs associated with oil content and mapping FAD2 genes and their relative contribution to oil quality in peanut (*Arachis hypogaea* L.). BMC Genet. 15, 133. 25491595 10.1186/s12863-014-0133-4 PMC4278341

[pbi12930-bib-0037] Pandey, M.K. , Roorkiwal, M. , Singh, V.K. , Ramalingam, A. , Kudapa, H. , Thudi, M. , Chitikineni, A. *et al* (2016) Emerging genomic tools for legume breeding: current status and future prospects. Front Plant Sci. 7, 455. 27199998 10.3389/fpls.2016.00455 PMC4852475

[pbi12930-bib-0038] Pandey, M.K. , Wang, H. , Khera, P. , Vishwakarma, M.K. , Kale, S.M. , Culbreath, A.K. , Holbrook, C.C. *et al* (2017a) Genetic dissection of novel QTLs for resistance to leaf spots and tomato spotted wilt virus in peanut (*Arachis hypogaea* L.). Front Plant Sci. 8, 25. 28197153 10.3389/fpls.2017.00025 PMC5281592

[pbi12930-bib-0039] Pandey, M.K. , Agarwal, G. , Kale, S.M. , Clevenger, J. , Nayak, S.N. , Sriswathi, M. , Chitikineni, A. *et al* (2017b) Development and evaluation of a high density genotyping ‘Axiom_Arachis’ array with 58 K SNPs for accelerating genetics and breeding in groundnut. Sci. Rep. 7, 40577. 28091575 10.1038/srep40577 PMC5238394

[pbi12930-bib-0040] Pandey, M.K. , Khan, A.W. , Singh, V.K. , Vishwakarma, M.K. , Shasidhar, Y. , Kumar, V. , Garg, V. *et al* (2017c) QTL‐seq approach identified genomic regions and diagnostic markers for rust and late leaf spot resistance in groundnut (Arachis hypogaea L.). Plant Biotechnol. J. 15, 927–941. 28028892 10.1111/pbi.12686 PMC5506652

[pbi12930-bib-0041] Patil, G. , Chaudhary, J. , Vuong, T.D. , Jenkins, B. , Qiu, D. , Kadam, S. , Shannon, G.J. *et al* (2017) Development of SNP genotyping assays for seed composition traits in soybean. Int. J. Plant. Genom. 2017, 12. 10.1155/2017/6572969 PMC5463095 28630621

[pbi12930-bib-0042] de Paula Santos Martins, C. , Pedrosa, A.M. , Du, D. , Gonçalves, L.P. , Yu, Q. , Gmitter Jr, F.G. , Costa, G.G. (2015) Genome‐wide characterization and expression analysis of major intrinsic proteins during abiotic and biotic stresses in sweet orange (*Citrus sinensis* L. Osb.). PLoS ONE, 10, e0138786. 26397813 10.1371/journal.pone.0138786 PMC4580632

[pbi12930-bib-0043] Qi, X. , Li, M.W. , Xie, M. , Liu, X. , Ni, M. , Shao, G. , Song, C. *et al* (2014) Identification of a novel salt tolerance gene in wild soybean by whole‐genome sequencing. Nat. Commun. 5, 4340. 25004933 10.1038/ncomms5340 PMC4104456

[pbi12930-bib-0044] Qin, H. , Feng, S. , Chen, C. , Guo, Y. , Knapp, S. , Culbreath, A. , He, G. *et al* (2012) An integrated genetic linkage map of cultivated peanut (*Arachis hypogaea* L.) constructed from two RIL populations. Theor. Appl. Genet. 124, 653–664. 22072100 10.1007/s00122-011-1737-y

[pbi12930-bib-0045] Romero, L.C. , Aroca, M.Á. , Laureano‐Marín, A.M. , Moreno, I. , García, I. and Gotor, C. (2014) Cysteine and cysteine‐related signaling pathways in *Arabidopsis thaliana* . Mol. Plant. 7, 264–276. 24285094 10.1093/mp/sst168

[pbi12930-bib-0046] Saxena, R.K. , Von Wettberg, E. , Upadhyaya, H.D. , Sanchez, V. , Songok, S. , Saxena, K. , Kimurto, P. *et al* (2014) Genetic diversity and demographic history of Cajanus spp. illustrated from genome‐wide SNPs. PLoS ONE, 9, e88568. 24533111 10.1371/journal.pone.0088568 PMC3922937

[pbi12930-bib-0047] Tadmor, Y. , Zamir, D. and Ladizinsky, G. (1987) Genetic mapping of an ancient translocation in the genus Lens. Theor. Appl. Genet. 73, 883–892. 24241299 10.1007/BF00289394

[pbi12930-bib-0048] Tang, D. , Wang, G. and Zhou, J.M. (2017) Receptor kinases in plant‐pathogen interactions: More than pattern recognition. Plant Cell, 29, 618–637. 28302675 10.1105/tpc.16.00891 PMC5435430

[pbi12930-bib-0049] Varshney, R.K. , Nayak, S.N. , May, G.D. and Jackson, S.A. (2009) Next‐generation sequencing technologies and their implications for crop genetics and breeding. Trends Biotechnol. 27, 522–530. 19679362 10.1016/j.tibtech.2009.05.006

[pbi12930-bib-0050] Varshney, R.K. , Mohan, S.M. , Gaur, P.M. , Gangarao, N.V.P.R. , Pandey, M.K. , Bohra, A. , Sawargaonkar, S.L. *et al* (2013) Achievements and prospects of genomics‐assisted breeding in three legume crops of the semi‐arid tropics. Biotechnol. Adv. 31, 1120–1134. 23313999 10.1016/j.biotechadv.2013.01.001

[pbi12930-bib-0051] Wang, H. , Lockwood, S.K. , Hoeltzel, M.F. and Schiefelbein, J.W. (1997) The ROOT HAIR DEFECTIVE3 gene encodes an evolutionarily conserved protein with GTP‐binding motifs and is required for regulated cell enlargement in Arabidopsis. Genes Dev. 11, 799–811. 9087433 10.1101/gad.11.6.799

[pbi12930-bib-0052] Wang, H. , Penmetsa, R.V. , Yuan, M. , Gong, L. , Zhao, Y. , Guo, B. , Farmer, A.D. *et al* (2012) Development and characterization of BAC‐end sequence derived SSRs, and their incorporation into a new higher density genetic map for cultivated peanut (*Arachis hypogaea* L.). BMC Plant Biol. 12, 10. 22260238 10.1186/1471-2229-12-10 PMC3298471

[pbi12930-bib-0053] Wang, H. , Pandey, M.K. , Qiao, L. , Qin, H. , Culbreath, A.K. , He, G. , Varshney, R.K. *et al* (2013) Genetic mapping and quantitative trait loci analysis for disease resistance using F_2_ and F_5_ generation‐based genetic maps derived from ‘Tifrunner’×’GT‐C20’ in peanut. Plant Genome, 6, 3.

[pbi12930-bib-0054] Wang, H. , Khera, P. , Huang, B. , Yuan, M. , Katam, R. , Zhuang, W. , Harris‐Shultz, K. *et al* (2016) Analysis of genetic diversity and population structure of peanut cultivars and breeding lines from China, India, and the US using simple sequence repeat markers. J. Int. Plant Biol. 58, 452–465. 10.1111/jipb.12380 26178804

[pbi12930-bib-0055] Xu, X. , Zeng, L. , Tao, Y. , Vuong, T. , Wan, J. , Boerma, R. , Noe, J. *et al* (2013) Pinpointing genes underlying the quantitative trait loci for root‐knot nematode resistance in palaeopolyploid soybean by whole genome resequencing. Proc. Nat. Acad. Sci. 110, 13469–13474. 23898176 10.1073/pnas.1222368110 PMC3746920

[pbi12930-bib-0056] Zhou, X. , Xia, Y. , Ren, X. , Chen, Y. , Huang, L. , Huang, S. , Liao, B. *et al* (2014) Construction of a SNP‐based genetic linkage map in cultivated peanut based on large scale marker development using next‐generation double‐digest restriction‐site‐associated DNA sequencing (ddRADseq). BMC Genom. 15, 351. 10.1186/1471-2164-15-351 PMC4035077 24885639

