# Supplementary figures and images for: High‐density genetic map using whole‐genome resequencing for fine mapping and candidate gene discovery for disease resistance in peanut
Source: Plant Biotechnol J. 2018 May 15;16(11):1954–67. doi: 10.1111/pbi.12930 (PMC6181220; doi:10.1111/pbi.12930)

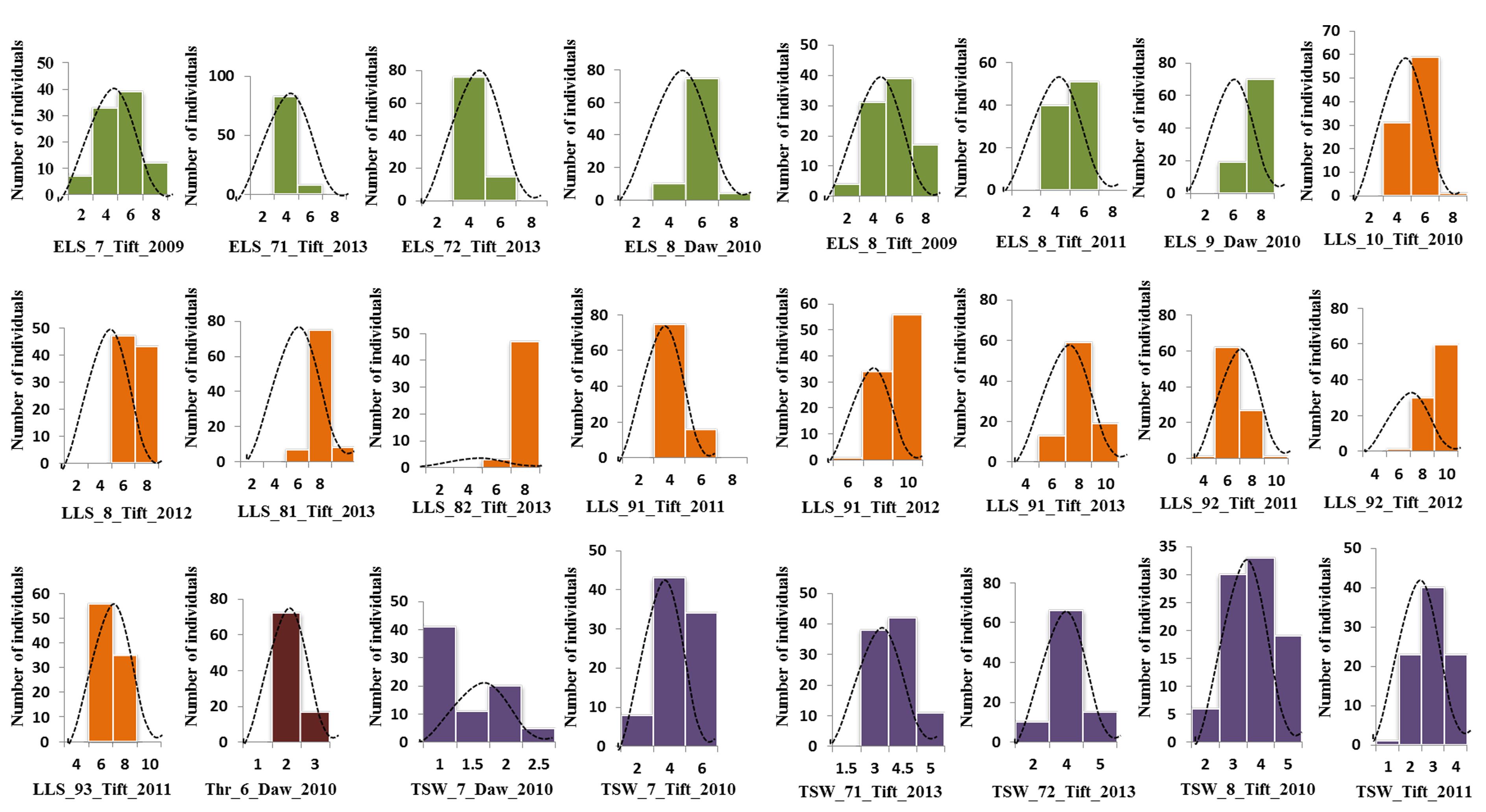

Supplement: Supplementary file 1 — Figure S1 Phenotypic distribution of ELS, LLS and TSWV in T‐pop RILs during different seasons. [file PBI-16-1954-s006.jpg]

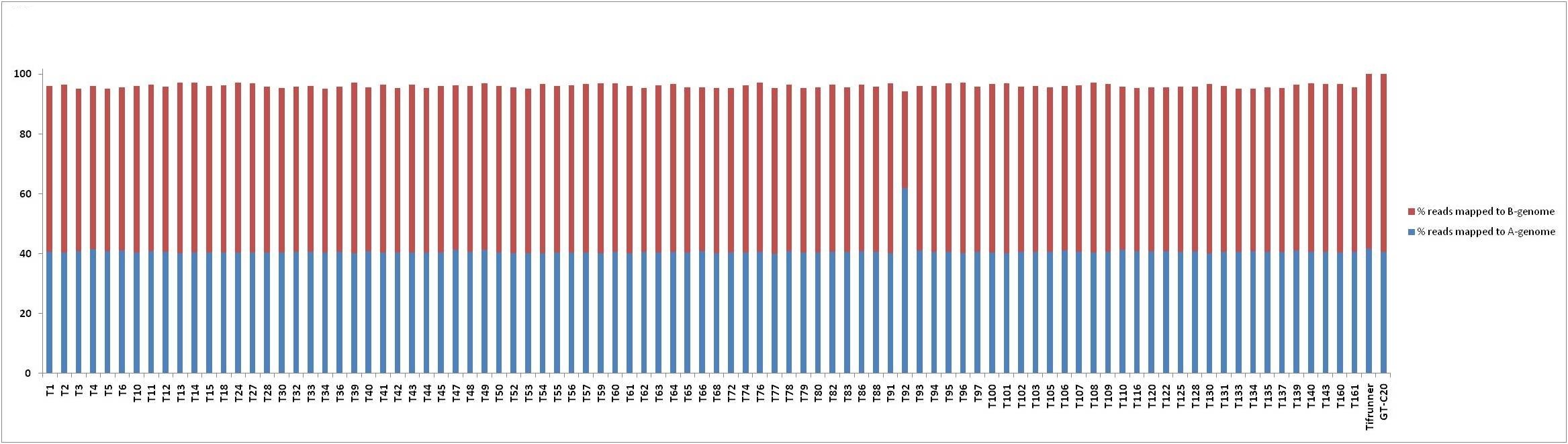

Supplement: Supplementary file 2 — Figure S2 Percentage reads mapped to the diploid reference A‐ and B‐genome in each RIL and the two parents. [file PBI-16-1954-s005.jpg]

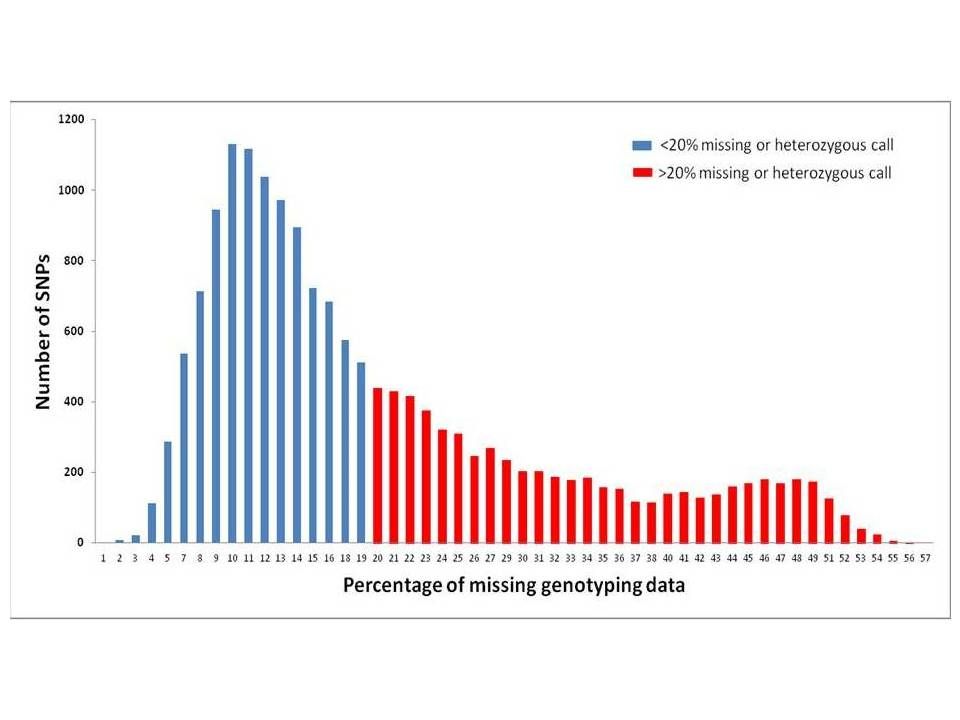

Supplement: Supplementary file 3 — Figure S3 Frequency histogram of the percentage of missing data points in WGRS of 91 RILs with 16 674 polymorphic SNPs in the population. [file PBI-16-1954-s004.jpg]

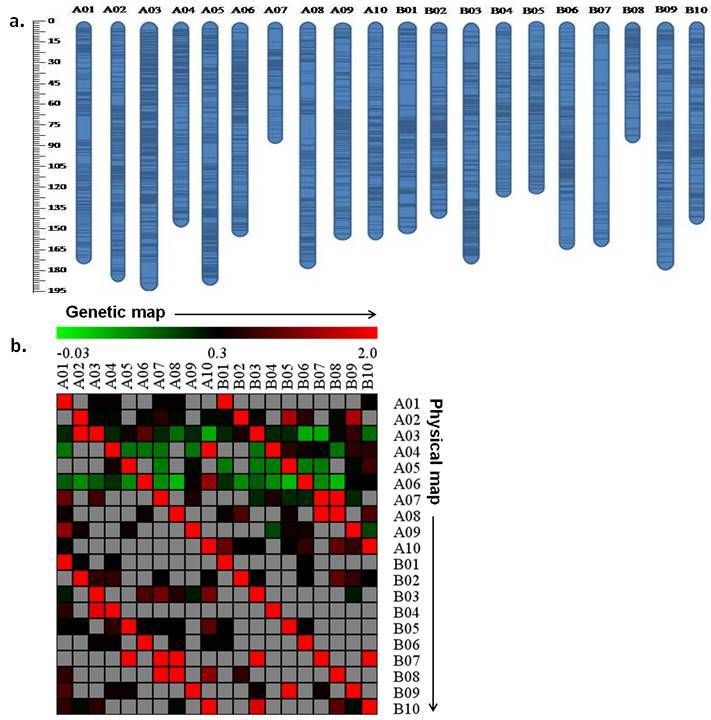

Supplement: Supplementary file 4 — Figure S4 Distribution of markers on linkage groups. [file PBI-16-1954-s003.jpg]

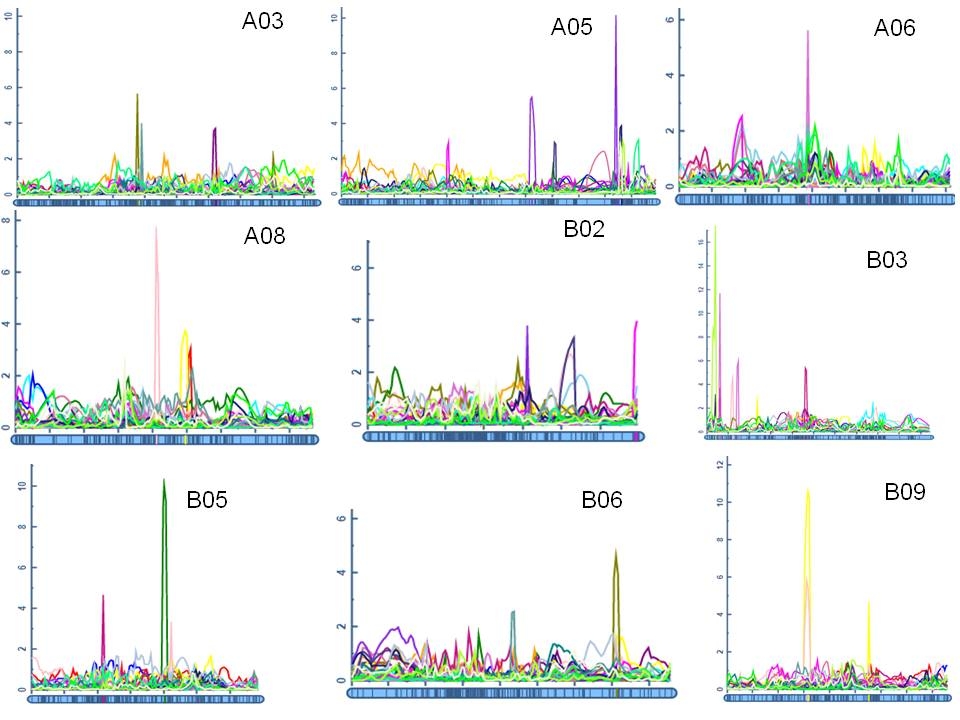

Supplement: Supplementary file 5 — Figure S5 QTL maps showing the major QTL peaks at different LODs on vertical axis. [file PBI-16-1954-s007.jpg]

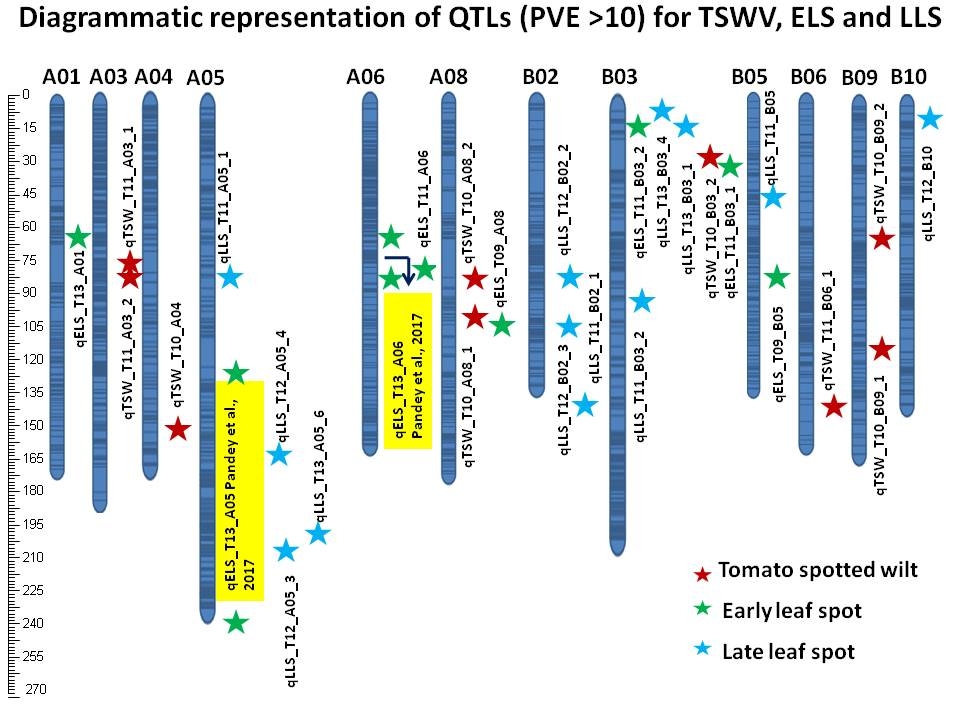

Supplement: Supplementary file 6 — Figure S6 Genetic and QTL map of major QTLs (>10% PVE) comprising SNP and SSR markers in Tifrunner × GT‐C20 population in peanut (Pandey et al., 2017a). [file PBI-16-1954-s002.jpg]
