# Supplementary material for: High‐density genetic map using whole‐genome resequencing for fine mapping and candidate gene discovery for disease resistance in peanut
Source: Plant Biotechnol J. 2018 May 15;16(11):1954–67. doi: 10.1111/pbi.12930 (PMC6181220; doi:10.1111/pbi.12930)
Supplement: Supplementary file 7 — Table S1 Phenotypic variation of diseases (ELS, LLS and TSWV) in T‐population parents and RILs. Table S2 Overview of the WGRS data and alignment to the reference genome. Table S3 Summary of SNPs detected between Tifrunner and GT‐C20 and SNPs used in RIL population. Table S4 Number of homeologus and translocated markers. Table S5 Effect of major QTL associated SSR markers reported in earlier study on the QTL and length of current genetic map. Table S6 Summary of environment QTLs detected in T‐population. Table S7 Summary of the epistatic QTLs detected across different environments and locations for the ELS, LLS and TSWV in the RILs. Table S8 SNPs in putative candidate genes underlying the major QTLs for ELS, LLS and TSWV resistance with their effect and impact. Table S9 SNP efficiency of markers validated using KASP assay. Table S10 List of primer sequences for KASP assay of SNPs developed and validated for Early Leaf Spot (ELS) and Late Leaf Spot (LLS). [file PBI-16-1954-s001.doc]

**Supplementary Tables**

**Table S1.** Phenotypic variation of diseases (ELS, LLS and TSWV) in T-population parents and RILs. Disease severity is on the scale of 1 to 10.

| **Year_month** | **Tifrunner** | **GT-C20** | **Mean** | **SD** | **CV (%)** | **Min** | **Max** |
| --- | --- | --- | --- | --- | --- | --- | --- |
| **ELS** | | | | | | | |
| 2009_July | 3 | 7 | 4.9 | 2.25 | 53.9 | 1 | 8 |
| 2009_Aug | 2.5 | 7 | 4.8 | 1.6 | 35.2 | 1.5 | 8 |
| 2011_Aug | 3.3 | 5.7 | 4.1 | 0.5 | 13.1 | 2.3 | 5.5 |
| 2013_July | 2.3 | 3.8 | 3.4 | 0.6 | 19.1 | 2.3 | 4.3 |
| **LLS** | | | | | | | |
| 2011_Sept | 5 | 7.2 | 5.9 | 0.4 | 7.3 | 4.7 | 7.3 |
| 2012_Sept | 5.2 | 9.3 | 8.3 | 0.4 | 4.7 | 5.2 | 10 |
| 2013_Aug | 6.5 | 10 | 7.8 | 0.4 | 5.1 | 4.5 | 10 |
| 2013_Sept | 5.8 | 9.8 | 7.3 | 0.6 | 7.8 | 4.8 | 10 |
| **TSWV** | | | | | | | |
| 2010_July | 1.8 | 5 | 3.8 | 1.3 | 37.6 | 1 | 6.7 |
| 2010_Aug | 2.3 | 5.7 | 3.5 | 1 | 28.4 | 1.7 | 6 |
| 2011_Aug | 1.7 | 4.7 | 2.8 | 0.79 | 28.2 | 2.3 | 4.3 |

**Table S2. Overview of the WGRS data and alignment to the reference genome.**

|  | **Total (millions)** |
| --- | --- |
| **Raw data of parents** |  |
| Reads Tifrunner | 6464.26 |
| Bases (bp) | 614790.32 |
| Reads GT-C20 | 427.05 |
| Bases (bp) | 42704.52 |
| **After filtering**  Filtered reads Tifrunner | 3453.64 |
| Bases (bp) | 329737.51 |
| Filtered reads GT-C20 | 352.44 |
| Bases (bp) | 33834.09 |
| **Mapped reads on reference genome**  Tifrunner reads mapped on A-genome | 1115.44 |
| Bases (bp) | 104014.89 |
| Tifrunner reads mapped on B-genome | 1562.44 |
| Bases (bp) | 145697.10 |
| GT-C20 reads mapped on A-genome | 110.81 |
| Bases (bp) | 10637.90 |
| GT-C20 reads mapped on B-genome | 161.92 |
| Bases (bp) | 15544.77 |
| **Raw data of RILs**  Reads | 12276.89 |
| Bases (bp) | 1620154.98 |
| **After filtering** |  |
| Reads | 10725.33 |
| Bases (bp) | 1240368.12 |
| **Mapped reads on reference genome** |  |
| Reads mapped on A-genome | 4474.07 |
| Bases (bp) | 433984.50 |
| Reads mapped on B-genome | 6080.30 |
| Bases (bp) | 589788.73 |

Bases (bp): number of bases was calculated by multiplying the total reads with an average read length.

**Table S3.** Summary of SNPs detected between Tifrunner and GT-C20 and SNPs used in RIL population.

| **Chromosome** | **SNPs b/w Tifrunner and GT-C20** | | **Genotyped SNPs in population** |
| --- | --- | --- | --- |
|  | **Detected** | **Filtered** |  |
| A01 | 4,730 | 707 | 635 |
| A02 | 4,916 | 827 | 670 |
| A03 | 10,328 | 2,246 | 1881 |
| A04 | 8,144 | 1,336 | 1174 |
| A05 | 8,132 | 1,394 | 1255 |
| A06 | 8,201 | 2,771 | 2430 |
| A07 | 6,118 | 788 | 777 |
| A08 | 4,916 | 531 | 517 |
| A09 | 7,796 | 1,096 | 1162 |
| A10 | 7,741 | 984 | 1095 |
| B01 | 2,068 | 469 | 488 |
| B02 | 2,704 | 598 | 559 |
| B03 | 2,924 | 787 | 625 |
| B04 | 2,722 | 496 | 448 |
| B05 | 3,257 | 697 | 641 |
| B06 | 2,528 | 749 | 568 |
| B07 | 2,468 | 261 | 197 |
| B08 | 2,379 | 384 | 368 |
| B09 | 2,361 | 568 | 576 |
| B10 | 3,138 | 563 | 608 |
| **Total** | **97,571** | **18,252** | **16,674** |

**Table S4.** Number of homeologus and translocated markers.

|  | **A01** | **A02** | **A03** | **A04** | **A05** | **A06** | **A07** | **A08** | **A09** | **A10** | **B01** | **B02** | **B03** | **B04** | **B05** | **B06** | **B07** | **B08** | **B09** | **B10** |
| --- | --- | --- | --- | --- | --- | --- | --- | --- | --- | --- | --- | --- | --- | --- | --- | --- | --- | --- | --- | --- |
| **A01** | - | 0 | 1 | 1 | 0 | 0 | 1 | 1 | 0 | 0 | 13 | 0 | 0 | 0 | 0 | 0 | 0 | 0 | 0 | 1 |
| **A02** | 0 | - | 1 | 1 | 0 | 1 | 2 | 1 | 0 | 1 | 1 | 27 | 1 | 0 | 5 | 2 | 0 | 1 | 5 | 0 |
| **A03** | 3 | 37 | - | 3 | 5 | 11 | 3 | 2 | 3 | 1 | 3 | 6 | 76 | 3 | 3 | 1 | 1 | 4 | 8 | 2 |
| **A04** | 1 | 0 | 0 | - | 1 | 1 | 1 | 0 | 1 | 26 | 0 | 0 | 1 | 33 | 4 | 3 | 2 | 1 | 4 | 4 |
| **A05** | 0 | 0 | 0 | 3 | - | 0 | 1 | 0 | 2 | 0 | 1 | 0 | 1 | 1 | 36 | 1 | 1 | 0 | 2 | 5 |
| **A06** | 3 | 2 | 3 | 4 | 2 | - | 2 | 1 | 6 | 22 | 4 | 2 | 3 | 2 | 1 | 46 | 2 | 1 | 5 | 5 |
| **A07** | 4 | 0 | 3 | 0 | 0 | 0 | - | 0 | 2 | 0 | 0 | 0 | 1 | 2 | 1 | 1 | 15 | 8 | 1 | 0 |
| **A08** | 1 | 0 | 0 | 0 | 0 | 1 | 0 | - | 0 | 0 | 1 | 2 | 0 | 0 | 1 | 0 | 13 | 13 | 0 | 2 |
| **A09** | 6 | 2 | 0 | 0 | 2 | 0 | 0 | 0 | - | 0 | 0 | 0 | 0 | 1 | 2 | 2 | 0 | 0 | 33 | 1 |
| **A10** | 1 | 0 | 0 | 0 | 0 | 0 | 0 | 0 | 0 | - | 3 | 1 | 1 | 0 | 1 | 2 | 0 | 3 | 2 | 25 |
| **B01** | 29 | 1 | 0 | 1 | 0 | 0 | 0 | 0 | 0 | 0 | - | 0 | 0 |  | 0 | 1 | 0 | 0 | 0 | 0 |
| **B02** | 0 | 37 | 2 | 2 | 0 | 0 | 1 | 0 | 0 | 1 | 0 | - | 1 | 0 | 0 | 1 | 0 | 3 | 2 | 1 |
| **B03** | 1 | 0 | 153 | 0 | 0 | 3 | 4 | 2 | 1 | 4 | 0 | 0 | - | 0 | 0 | 0 | 0 | 0 | 1 | 0 |
| **B04** | 1 | 0 | 3 | 26 | 0 | 0 | 0 | 0 | 0 | 0 | 0 | 0 | 0 | - | 0 | 0 | 0 | 0 | 0 | 0 |
| **B05** | 1 | 1 | 0 | 2 | 40 | 1 | 1 | 1 | 0 | 3 | 1 | 0 | 0 | 0 | - | 1 | 0 | 0 | 0 | 0 |
| **B06** | 0 | 0 | 1 | 1 | 0 | 41 | 0 | 1 | 0 | 1 | 1 | 0 | 0 | 0 | 0 | - | 0 | 0 | 0 | 0 |
| **B07** | 0 | 0 | 0 | 0 | 1 | 0 | 6 | 4 | 0 | 0 | 0 | 0 | 3 | 0 | 0 | 0 | - | 0 | 0 | 1 |
| **B08** | 1 | 0 | 0 | 0 | 0 | 0 | 12 | 8 | 0 | 2 | 0 | 1 | 0 | 0 | 0 | 0 | 0 | - | 0 | 0 |
| **B09** | 2 | 0 | 0 | 1 | 1 | 0 | 0 | 0 | 28 | 0 | 0 | 0 | 1 | 0 | 14 | 0 | 1 | 0 | - | 0 |
| **B10** | 2 | 1 | 2 | 0 | 0 | 0 | 0 | 1 | 0 | 54 | 0 | 0 | 6 | 0 | 0 | 0 | 0 | 3 | 1 | - |

Highlighted in grey and yellow are the homologous markers for A- and B- LGs respectively. Numbers other than the highlighted ones depict distribution of the translocated SNP markers.

**Table S5.** Effect of major QTL associated SSR markers reported in earlier study (Pandey et al., 2017a) on the QTL and length of current genetic map. The table shows the difference in length, LOD values and PVE% of the QTLs identified using the SSRs in earlier study and using the SNPs in the current study.

| **Marker name (SSR)** | **LG^1^** | **LG^2^** | **No. of markers^a^** | **No. of markers^b^** | **Length^a^ (cM )** | **Length^b^ (cM )** | **Trait** | **LOD^1^** | **LOD^2^** | **PVE^1^** | **PVE^2^** |
| --- | --- | --- | --- | --- | --- | --- | --- | --- | --- | --- | --- |
| GA27, GM2388 | A03 | B03 | 373 | 375 | 175.91 | 208.19 | ELS | 3.3 | 2.9 | 11.5 | 12.6 |
| GM1062, TC23B10 | A04 | A04 | 676 | 678 | 153.52 | 181.96 | TSWV | 6.3 | _ | 14.4 | _ |
| TC40D04, GM1878, GNB703, PM65, PM179 | A05 | A05 | 701 | 706 | 190.24 | 240.54 | ELS, LLS | 4 - 5.2 | 2.8-9.8 | 11.2-15.1 | 10.2-47.6 |
| Sseq18G9-1, TC28E09 | A06 | A06 | 1644 | 1646 | 157.13 | 172.95 | ELS | 5.7 | 5.2 | 13.2 | 17.4 |
| TC38F01, GM1986-2 | A07 | A07 | 406 | 408 | 89.59 | 119.62 | LLS | 3.8 | _ | 12.8 | _ |
| AHGS0590, TC38D06-1 | B06 | B06 | 283 | 285 | 163.98 | 174.49 | ELS | 3.3 | _ | 10.6 | _ |
| **Total** |  |  | **4083** | **4098** | **930.37** | **1097.75** |  |  |  |  |  |

^1,2^ represent the earlier and current LGs, number of markers, length of genetic maps, LOD values and PVE% obtained for the traits associated with those markers. ^a,b^ represent the number of markers and length of the current genetic map before and after addition of SSRs. The two SSR markers that were earlier mapped on A03 were mapped on LG B03 in the current study. Rest of the SSRs was mapped on the same LG. – represent that no QTL was identified associated with SNPs between the SSRs in this study

**Table S6. Summary of environment QTLs detected in T-population.**

| **Trait** | **Chromosome** | **Position** | **Left Marker** | **Right Marker** | **LOD (AbyE)** | **PVE (AbyE)** | **Add** |
| --- | --- | --- | --- | --- | --- | --- | --- |
| ELS | B05 | 80 | B05_22527171 | B05_22119840 | 3.60 | 11.89 | -0.20 |
|  | A08 | 102 | A08_35585793 | A08_35776787 | 2.53 | 4.95 | 0.10 |
|  | B03 | 12 | A03_133651613 | A03_133731756 | 12.95 | 3.75 | -0.06 |
|  | B09 | 94 | B09_10577428 | B09_9939719 | 2.18 | 3.00 | -0.07 |
|  | A04 | 160 | B09_135356276 | A04_294352 | 2.84 | 2.82 | -0.04 |
|  | B04 | 82 | B04_12680096 | B04_11021922 | 2.64 | 2.67 | 0.08 |
|  | A08 | 66 | A08_20471293 | A08_25723467 | 3.37 | 2.66 | 0.03 |
| LLS | B03 | 8 | B03_135471057 | B03_867854 | 8.79 | 5.95 | -0.21 |
|  | A05 | 208 | A05_20406182 | B05_20992208 | 8.79 | 4.82 | -0.07 |
|  | A05 | 144 | A05_82270000 | A05_54130650 | 4.79 | 2.17 | 0.08 |
| TSWV | B09 | 63 | B09_6739506 | A09_5826749 | 9.00 | 8.35 | 0.22 |
|  | B09 | 110 | A09_9631598 | B09_14497666 | 4.48 | 3.42 | -0.10 |
|  | A08 | 99 | A08_35464654 | A08_35813151 | 3.01 | 2.59 | 0.12 |

**Table S7.** Summary of the epistatic QTLs detected across different environments and locations for the ELS, LLS and TSWV in the RILs

|  |  | |  | | |  |
| --- | --- | --- | --- | --- | --- | --- |
| **Traits** | | **No of QTL-QTL interactions** | | **PVE range** | **Total PVE explained by all interactions** | |
| **Early leaf spot** | |  | |  |  | |
| ELS_7_Tift_2009 | | 42 | | 9.7-21.8 | 22.86 | |
| ELS_71_Tift_2013 | | 43 | | 9.8-24.4 | 23.18 | |
| ELS_72_Tift_2013 | | 81 | | 10.3-23.2 | 13.48 | |
| ELS_8_Daw_2010 | | 44 | | 8.2-16.2 | 35.37 | |
| ELS_8_Tift_2009 | | 42 | | 9.1-18.9 | 27.39 | |
| ELS_8_Tift_2011 | | 60 | | 5.1-11.5 | 59.43 | |
| ELS_9_Daw_2010 | | 48 | | 13.2-27.9 | 0.00 | |
| **Late leaf spot** | |  | |  |  | |
| LLS_10_Tift_2010 | | 58 | | 13.2-29.3 | 0.00 | |
| LLS_8_Tift_2012 | | 59 | | 8.5-17.1 | 32.73 | |
| LLS_81_Tift_2013 | | 65 | | 10.4-21.1 | 15.99 | |
| LLS_82_Tift_2013 | | 57 | | 6.5-14.9 | 36.46 | |
| LLS_91_Tift_2011 | | 32 | | 6.6-14.4 | 44.91 | |
| LLS_91_Tift_2012 | | 61 | | 8.1-22.3 | 33.44 | |
| LLS_91_Tift_2013 | | 52 | | 9.2-21.2 | 23.22 | |
| LLS_92_Tift_2011 | | 42 | | 11.3-21.6 | 0.00 | |
| LLS_92_Tift_2012 | | 51 | | 7.5-14.8 | 38.46 | |
| LLS_93_Tift_2011 | | 28 | | 8.8-17.9 | 29.75 | |
| **Tomato spotted wilt virus** | |  | |  |  | |
| TSW_7_Daw_2010 | | 58 | | 10.9-40.6 | 12.59 | |
| TSW_7_Tift_2010 | | 66 | | 6.9-18.8 | 40.63 | |
| TSW_71_Tift_2013 | | 44 | | 11.3-21.1 | 16.59 | |
| TSW_72_Tift_2013 | | 55 | | 9.3-23.9 | 27.33 | |
| TSW_8_Tift_2010 | | 51 | | 5.2-16.2 | 55.30 | |
| TSW_Tift_2011 | | 59 | | 6.8-25.8 | 45.07 | |

**Table S8.** SNPs in putative candidate genes underlying the major QTLs for ELS, LLS and TSWV resistance with their effect and impact.

| **Gene** | **SNP position** | **Chr** | **Tif base** | **C-20 base** | **Trait** | **Intron/ Exon/UTR** | **Function** | **Effect** | | **Impact** |
| --- | --- | --- | --- | --- | --- | --- | --- | --- | --- | --- |
| Aradu.4I7WA^*^ | 133486715 | A03 | G | A | ELS | Exon | Xyloglucan endotransglucosylase/hydrolase | Start lost | | High (Missense) |
| Aradu.VB4ZI^*^ | 133511189 | A03 | T | C | ELS | Exon | UDP-Glycosyltransferase superfamily protein | Non synonymous | | Moderate (Missense) |
| Aradu.VB4ZI^*^ | 133511926 | A03 | G | C | ELS | Exon | UDP-Glycosyltransferase superfamily protein | Synonymous | | Low |
| Aradu.VB4ZI^*^ | 133515559 | A03 | C | T | ELS | Intron | UDP-Glycosyltransferase superfamily protein | _ | | _ |
| Aradu.031BD^*^ | 133598286 | A03 | C | G | ELS | 5’ UTR | Unknown | UTR 5' modifier | |  |
| Aradu.HU6GA^*^ | 133636612 | A03 | A | G | ELS | Exon | S-adenosyl-L-homocysteine hydrolase | Synonymous | | Low (Silent) |
| Aradu.C56U2 | 133693128 | A03 | T | A | ELS | Intron | Rhodanese/cell cycle control phosphatase superfamily protein | _ | | _ |
| Aradu.C56U2 | 133693866 | A03 | G | T | ELS | Intron | Rhodanese/cell cycle control phosphatase superfamily protein | _ | | _ |
| Aradu.C56U2 | 133693873 | A03 | T | C | ELS | Intron | Rhodanese/cell cycle control phosphatase superfamily protein | _ | | _ |
| Aradu.0P6EN^*^ | 133799472 | A03 | C | G | ELS | Intron | Methyltransferase like protein | _ | | _ |
| Aradu.0P6EN^*^ | 133803373 | A03 | G | A | ELS | Exon | Methyltransferase like protein | Synonymous | | Low (Silent) |
| Aradu.N20HG^*^ | 133826323 | A03 | T | A | ELS | Exon | ATP/DNA-binding protein | Synonymous | | Low (Silent) |
| Aradu.453WH^*^ | 133841579 | A03 | G | A | ELS | Intron | Small ubiquitin-like modifier | _ | | _ |
| Aradu.453WH^*^ | 133841848 | A03 | A | T | ELS | 5’ UTR | Small ubiquitin-like modifier | UTR 5' modifier | | _ |
| Araip.C8K92 | 22527174 | B05 | C | T | ELS | Intron | Unknown | _ | | _ |
| Araip.C1BT9 | 20207815 | B05 | C | G | ELS | Exon | Cell wall protein involved in cellulose microfibril organization | Non synonymous | | Moderate (Missense) |
| Araip.A06C0 | 20375522 | B05 | A | G | ELS | Intron | Gibberellin 2-beta-dioxygenase 8-like | _ | | _ |
| Araip.A06C0 | 20376369 | B05 | C | T | ELS | 3’ UTR | Gibberellin 2-beta-dioxygenase 8-like | UTR 3' modifier | | _ |
| Araip.61FFX | 20673655 | B05 | G | A | ELS | Intron | Phosphotransferases | _ | | _ |
| Araip.61FFX | 20693095 | B05 | C | T | ELS | Exon | Phosphotransferases | Non synonymous | | Moderate (Missense) |
| Araip.8IP9E | 20700172 | B05 | C | A | ELS | Intron | Inositol-tetrakisphosphate 1-kinase | _ | | _ |
| Araip.GC5DY | 21207791 | B05 | A | T | ELS | Intron | Uncharacterized | _ | | _ |
| Araip.CF28G | 21249724 | B05 | T | G | ELS | Intron | Pathogenesis-related homeodomain protein isoform X1 | _ | | _ |
| Araip.CF28G | 21249734 | B05 | G | C | ELS | Intron | Pathogenesis-related homeodomain protein isoform X1 | _ | | _ |
| Araip.3BJ9Q | 21293668 | B05 | A | T | ELS | Intron | DNA repair protein | _ | | _ |
| Araip.IHB0P | 21418717 | B05 | G | A | ELS | Exon | Spindle pole body component | Non synonymous | | Moderate (Missense) |
| Araip.UC596 | 21678054 | B05 | G | T | ELS | Intron | Multi antimicrobial extrusion protein (MATE efflux family protein) | _ | | _ |
| Araip.A3ZSJ | 21875174 | B05 | G | T | ELS | Intron | Uncharacterized protein | _ | | _ |
| Araip.IX7QU | 22093849 | B05 | G | A | ELS | 3’ UTR | Protein kinase superfamily protein | UTR 3' modifier | | _ |
| Araip.C8K92 | 22527171 | B05 | T | C | ELS | Intron | Unknown | _ | | _ |
| Aradu.VP5WD | 134282530 | A03 | T | C | LLS | Intron | Myb family transcription factor | _ | | _ |
| Aradu.VP5WD | 134283359 | A03 | G | C | LLS | Intron | Myb family transcription factor | _ | | _ |
| Aradu.YL3ZN | 134333508 | A03 | G | C | LLS | Exon | Receptor-like kinase | Synonymous | | Low |
| Aradu.RTW4C | 134474041 | A03 | C | A | LLS | Exon | Uncharacterized | Synonymous | | Low (Silent) |
| Aradu.V4NFM | 134502638 | A03 | T | G | LLS | Intron | Glutathione S-transferase family protein | _ | | _ |
| Aradu.V4NFM | 134513642 | A03 | T | G | LLS | Intron | Glutathione S-transferase family protein | _ | | _ |
| Aradu.3N55U | 134555474 | A03 | T | G | LLS | Exon | Unknown | Synonymous | | Low |
| Aradu.3N55U | 134555617 | A03 | C | G | LLS | Exon | Unknown | Synonymous | | Low |
| Aradu.RVF1V | 134565498 | A03 | A | C | LLS | Intron | Heat shock transcription factor | _ | | _ |
| Aradu.VG4AF | 134604120 | A03 | T | C | LLS | Intron | Major intrinsic protein (MIP) family transporter | _ | | _ |
| Aradu.1JP2B | 20624528 | A05 | G | A | LLS | Intron | Phosphotransferase alcohol group as acceptor binding inositol or phosphatidylinositol kinases | _ | | _ |
| Aradu.1JP2B | 20643222 | A05 | C | T | LLS | Exon | Phosphotransferase alcohol group as acceptor binding inositol or phosphatidylinositol kinases | Non synonymous | | Moderate (Missense) |
| Aradu.GF39G | 20808068 | A05 | C | T | LLS | Intron | Ribonucleoside-diphosphate reductase | _ | | _ |
| Aradu.8YR6B | 21112744 | A05 | A | T | LLS | Intron | Pathogenesis-related homeodomain protein isoform X1 | _ | _ | |
| Aradu.N8CMN | 21135367 | A05 | T | A | LLS | Intron | Unknown | _ | _ | |
| Aradu.J0IE9 | 21137635 | A05 | T | A | LLS | Intron | Unknown | _ | _ | |
| Aradu.J0IE9 | 21137640 | A05 | C | G | LLS | Intron | Unknown | _ | _ | |
| Aradu.50UH7 | 21751497 | A05 | G | A | LLS | Intron | Unknown | _ | _ | |
| Aradu.666C5 | 21771336 | A05 | G | T | LLS | Intron | Multi antimicrobial extrusion protein (MATE efflux family protein) | _ | _ | |
| Aradu.GP9E2 | 21830237 | A05 | C | T | LLS | Intron | Uncharacterized protein | _ | _ | |
| Aradu.EJ66Y | 21868375 | A05 | G | A | LLS | Intron | Multi antimicrobial extrusion protein (MATE efflux family protein) | _ | _ | |
| Aradu.14QPB | 21989268 | A05 | G | T | LLS | Intron | Unknown | _ | _ | |
| Aradu.14QPB | 21993802 | A05 | C | T | LLS | Intron | Unknown | _ | _ | |
| Aradu.4K5XY | 22106785 | A05 | A | G | LLS | Exon | Alanine aminotransferase | Synonymous | Low | |
| Aradu.4K5XY | 22109697 | A05 | G | T | LLS | 5’ UTR | Alanine aminotransferase | UTR 5' modifier | _ | |
| Aradu.3QU7E | 22153404 | A05 | T | C | LLS | Exon | Unknown | Synonymous | Low | |
| Aradu.5UB6E | 22251051 | A05 | G | A | LLS | Intron | Tryptophan aminotransferase | _ | _ | |
| Aradu.5UB6E | 22252419 | A05 | G | T | LLS | Exon | Tryptophan aminotransferase | Synonymous | Low | |
| Araip.EC6NQ | 5189529 | B09 | C | T | TSWV | Exon | Disease resistance | Non synonymous | Moderate (Missense) | |
| Araip.A3IF2 | 5191304 | B09 | C | A | TSWV | Exon | Disease resistance | Non synonymous | Moderate (Missense) | |
| Araip.RN7PY | 5371184 | B09 | A | C | TSWV | Exon | Protein kinase family protein | Synonymous | Low (Silent) | |
| Araip.RN7PY | 5371237 | B09 | G | A | TSWV | Exon | Protein kinase family protein | Non synonymous | Moderate (Missense) | |
| Araip.RN7PY | 5372331 | B09 | G | C | TSWV | Intron | Protein kinase family protein | _ | _ | |
| Araip.MWI6K | 5401776 | B09 | T | C | TSWV | Exon | NB-ARC and LRR disease resistance protein (Defense response) | Non synonymous | Moderate (Missense) | |
| Araip.FIQ6P | 5436965 | B09 | A | C | TSWV | Intron | Protein kinase family protein | _ | _ | |
| Araip.8R852 | 5450124 | B09 | G | T | TSWV | Exon | Receptor protein kinase | Synonymous | Low (Silent) | |
| Araip.0S6VM | 5493962 | B09 | C | G | TSWV | Exon | Protein kinase family protein | Non synonymous | Moderate (Missense) | |
| Araip.FV54T | 5564544 | B09 | T | A | TSWV | Intron | Protein kinase family protein | _ | _ | |
| Araip.J0YZM | 5654471 | B09 | T | A | TSWV | Intron | Rho termination factor | _ | _ | |
| Araip.T9L48 | 5711119 | B09 | C | T | TSWV | Intron | IAA-amino acid hydrolase | _ | _ | |
| Araip.TB50A | 5755785 | B09 | C | G | TSWV | Exon | IAA-amino acid hydrolase ILR1-like protein | Non synonymous | Moderate (Missense) | |
| Araip.TB50A | 5756692 | B09 | A | G | TSWV | Intron | IAA-amino acid hydrolase ILR1-like protein | _ | _ | |
| Araip.SR3J8 | 5849967 | B09 | C | A | TSWV | Intron | Unknown | _ | _ | |
| Araip.5P4LE | 5921036 | B09 | T | A | TSWV | Intron | Ribosomal L-28 like protein | _ | _ | |
| Araip.5P4LE | 5921901 | B09 | A | T | TSWV | Exon | Ribosomal L-28 like protein | Synonymous | Low (Silent) | |
| Araip.WW8Y7 | 5935108 | B09 | A | G | TSWV | Exon | Glutamate dehydrogenase | Non synonymous | Moderate (Missense) | |
| Araip.9T22U | 6008567 | B09 | A | T | TSWV | Intron | F-box/RNI-like superfamily protein | _ | _ | |
| Araip.GL0PH | 6154301 | B09 | G | T | TSWV | Exon | Serine acetyltransferase | Non synonymous | Moderate (Missense) | |
| Araip.A7SD4 | 6174677 | B09 | C | T | TSWV | Intron | F-box/RNI-like superfamily protein | _ | _ | |
| Araip.M4KHY | 6361776 | B09 | T | C | TSWV | Intron | FBD-associated F-Box protein | _ | _ | |
| Araip.4M4DY | 6385121 | B09 | C | T | TSWV | Exon | FBD-associated F-Box protein | Non synonymous | Moderate (Missense) | |
| Araip.W3YFG | 6610053 | B09 | A | C | TSWV | Intron | HAD-superfamily hydrolase | _ | _ | |
| Araip.S85MS | 6642104 | B09 | A | T | TSWV | Intron | Root hair defective 3 homolog 1-like | _ | _ | |

* signify the SNPs within the genes 200 Kb up- and downstream

**Table S9.** SNP efficiency of markers validated using KASP assay. Concordant allele calls were the ones which confirmed the in-silico SNP call in the KASP validation. Discordant were the ones which showed mismatch of in-silico and KASP genotyping. Overall, the SNP efficiency was over 90% for each SNP. Some of the markers could not be assigned any genotype in KASP assay were therefore considered not validated.

|  | **Concordant alleles** | | | **Discordant alleles** | | | **Not validated** | **SNP efficiency (validation %)** |
| --- | --- | --- | --- | --- | --- | --- | --- | --- |
|  | **AA** | **BB** | **AB** | **AA** | **BB** | **AB** |  |  |
| A03_134198144 | 35 | 41 | 3 | 2 | 2 | 2 | 6 | 92.94 |
| A06_14301316 | 43 | 33 | 2 | 2 | 2 | 3 | 6 | 91.76 |
| A05_82270000 | 35 | 40 | 6 | 0 | 2 | 2 | 6 | 95.29 |
| B05_22527171 | 38 | 40 | 2 | 2 | 1 | 0 | 8 | 96.39 |
| A05_20406182 | 41 | 40 | 1 | 0 | 1 | 1 | 7 | 97.62 |

AA represent homozygous markers from the resistant parent (Tifrunner); BB represent the homozygous markers from the susceptible parent (GT-C20); AB represent the rare heterozygous call

**Table S10**. List of primer sequences for KASP assay of SNPs developed and validated for Early Leaf Spot (ELS) and Late Leaf Spot (LLS).

| ID | Trait | Primer_Tif_Allele | Primer_C-20_Allele | Primer_Common | ‘Tifrunner’ allele | ‘GT-C20’ allele |
| --- | --- | --- | --- | --- | --- | --- |
| A03_134198144 | LLS | AGATGCTGCACTACACTTTACTAATAC | AGATGCTGCACTACACTTTACTAATAG | GCCTCTTGAAAGTGAAGTTTGTGTGAATA | G | C |
| A05_20406182^#^ | LLS | AGTAAGGTGAAAGGTGATGGGTGAA | AGTAAGGTGAAAGGTGATGGGTGAT | CCAATTATCCATCCTTATCCTAATTCAGAT | A | T |
| A05_82270000^#^ | LLS | TCCATTCTAGCTACCGGGAT | CTTCCATTCTAGCTACCGGGAC | GCTCAAGGTTATGATCAAGAGGAAGAAAT | C | T |
| A06_14301316 | ELS | AACTTCTGTCAATCGAGTCTTAACTG | CAACTTCTGTCAATCGAGTCTTAACTA | TCTGCATCCATTGCCTGTTTTCAATGTA | T | C |
| B05_22527171^#^ | ELS | ACCAAAAAAGGTATTTACTCTATTTACGTG | GACCAAAAAAGGTATTTACTCTATTTACGTA | CTACACATGCAACCAAACCTTCTTCAAAT | T | C |

#SNPs associated with both major QTL and e-QTL
